# AdaLiftOver: high-resolution identification of orthologous regulatory elements with Adaptive liftOver

**DOI:** 10.1093/bioinformatics/btad149

**Published:** 2023-04-02

**Authors:** Chenyang Dong, Siqi Shen, Sündüz Keleş

**Affiliations:** Department of Statistics, University of Wisconsin-Madison, 1300 University Avenue, Madison, WI 53706, USA; Department of Biostatistics and Medical Informatics, University of Wisconsin-Madison, WARF Room 201, 610 Walnut Street, Madison, WI 53706, USA; Department of Statistics, University of Wisconsin-Madison, 1300 University Avenue, Madison, WI 53706, USA; Department of Biostatistics and Medical Informatics, University of Wisconsin-Madison, WARF Room 201, 610 Walnut Street, Madison, WI 53706, USA

## Abstract

**Motivation:**

Elucidating functionally similar orthologous regulatory regions for human and model organism genomes is critical for exploiting model organism research and advancing our understanding of results from genome-wide association studies (GWAS). Sequence conservation is the *de facto* approach for finding orthologous non-coding regions between human and model organism genomes. However, existing methods for mapping non-coding genomic regions across species are challenged by the multi-mapping, low precision, and low mapping rate issues.

**Results:**

We develop Adaptive liftOver (AdaLiftOver), a large-scale computational tool for identifying functionally similar orthologous non-coding regions across species. AdaLiftOver builds on the UCSC liftOver framework to extend the query regions and prioritizes the resulting candidate target regions based on the conservation of the epigenomic and the sequence grammar features. Evaluations of AdaLiftOver with multiple case studies, spanning both genomic intervals from epigenome datasets across a wide range of model organisms and GWAS SNPs, yield AdaLiftOver as a versatile method for deriving hard-to-obtain human epigenome datasets as well as reliably identifying orthologous loci for GWAS SNPs.

**Availability and implementation:**

The R package and the data for AdaLiftOver is available from https://github.com/keleslab/AdaLiftOver.

## 1 Introduction

Genome-wide association studies (GWAS) have revealed many non-coding SNPs for complex human traits (Welter et al. 2014, Gallagher and Chen-Plotkin 2018). However, identifying the effector genes of non-coding SNPs and elucidating their specific roles in disease etiologies are key challenges of modern GWAS. Model organism studies are important and under-exploited resources for dissecting GWAS SNPs by experimentally perturbing the orthologous model organism loci for the human genomic regions of interest. Reliable maps of non-coding genomic regions between human and model organism genomes will not only improve our understanding of the evolution of regulatory mechanisms but also pinpoint functionally similar regulatory elements for comparative genomics and epigenomics analysis.

**Figure 1:**
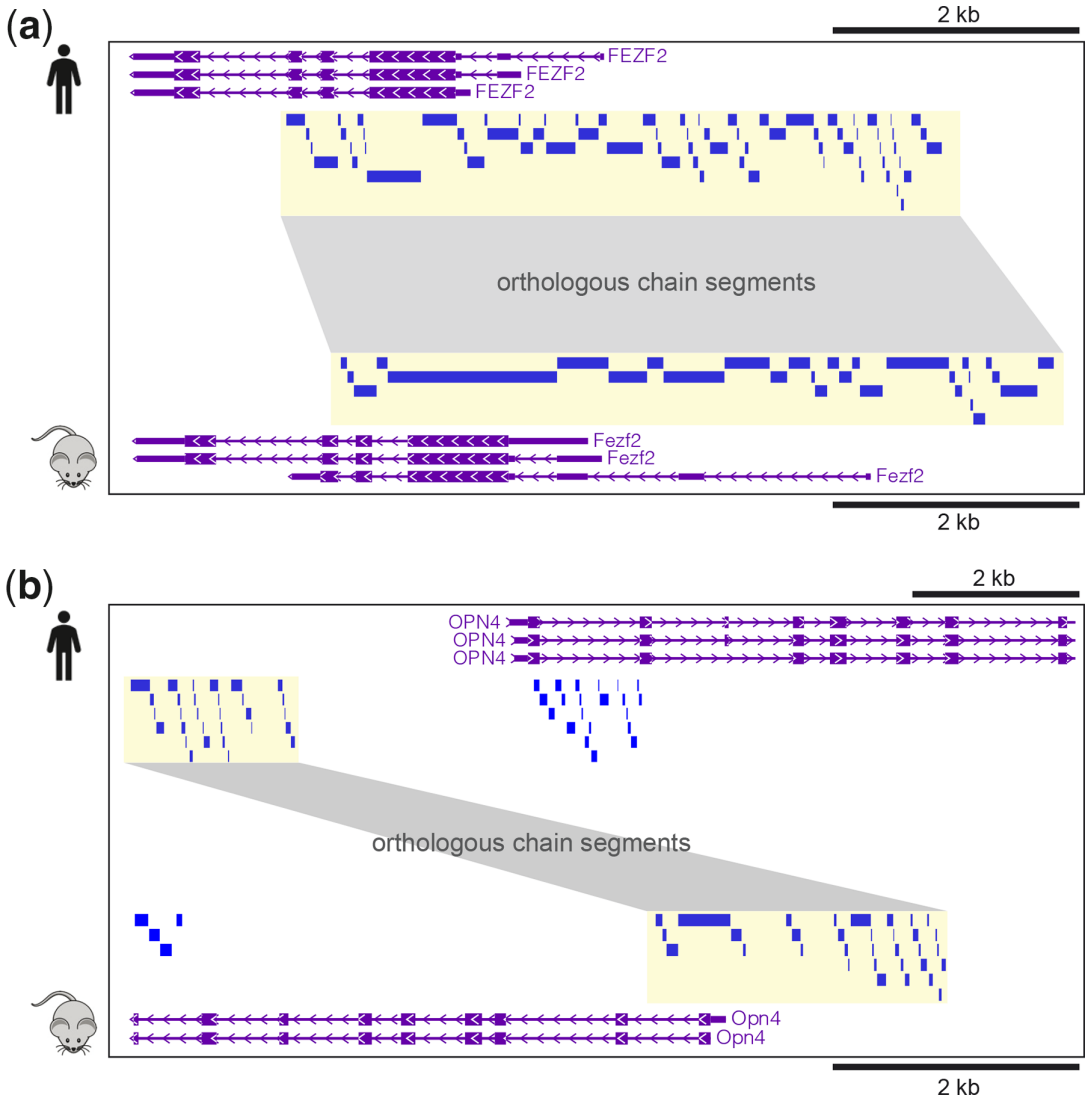
(a) The promoter of *FEZF2* in human maps directly to the promoter of *Fezf2* in mouse. (b) The promoter of *OPN4* in human maps indirectly to the promoter *Opn4* in mouse by allowing for a local window. The small rectangles depict chain segments from the pairwise sequence alignment. Orthologous chain segments between human and mouse are highlighted and connected with bands

Sequence alignment has made fundamental contributions to phylogenetic analysis and evolutionary biology (Earl et al. 2014). Leveraging DNA sequences as the mapping units is the standard approach to establish putative orthologous mappings across different species. The current architecture of translating genomic coordinates across genome assemblies is largely based on UCSC’s chained and netted sequence alignment results, which are summarized as chain files. The UCSC liftOver tool (Hinrichs et al. 2006) is the *de facto* mapping strategy in cross-species studies. More recently, bnMapper (Denas et al. 2015), which is a Python implementation similar to UCSC liftOver but leverages reciprocal chain files allowing for only one-to-one mappings across species has emerged. However, there are a number of practical drawbacks of these strictly sequence alignment-based mapping approaches of non-coding sequences. We group these into three categories as follows using the mappings between promoters of orthogolous human and mouse genes:

1. *The prevailing multi-mapping issues.* A given non-coding region in the human genome can be mapped, i.e. lifted over, to multiple mouse regions. For example, when we consider the 16 374 human genes with mouse orthologues, 94.7% of their promoters map to multiple mouse regions with an average of 38.8 ± 22.0 regions (Supplementary Section 1.1). Merging of the small gaps <10 bp yields mapping of the human promoters to an average of 3.68 ± 2.73 mouse regions and still leaves 78.6% as mapping to multiple regions. In particular, 34.8% of the human promoters map to multiple mouse regions separated apart by at least 200 bp.
2. Inaccurate mappings and low precision issues. Sequence-based mapping of the 16 374 orthologous human and mouse promoters is prone to generating 2842 (17.4%) false positive and 205 (1.2%) false negative cases (Supplementary Table S1). Figure 1a illustrates an example of a true positive (76.3% of all orthologous promoters) at the promoter regions of *FEZF2* in human and *Fezf2* in mouse. Figure 1b demonstrates a potential discrepancy between the orthologous chain segments at the promoter regions of *OPN4* and *Opn4*. As a consequence, UCSC liftOver and bnMapper fail to map the *OPN4* promoter to mouse genome. This promoter region can be mapped to the correct counterpart after extension on both sides and, hence, is classified as a false negative for the purposes of this exploration (Supplementary Section 1.1).
3. *Low mapping rates.* Unlike the highly conserved orthologous promoters, Cheng et al. (2014) found that ∼50% of the transcription factor occupied regions failed to map to the mouse genome and Dong et al. (2021) observed that ∼80% of diabetes related human GWAS SNPs were unmappable to the mouse genome. This highlights the general challenge of mapping human non-coding regions to model organism genomes.

Recent deep learning applications have yielded advanced investigations of the regulatory code of DNA sequences. Early applications of deep convolutional neural network models used only DNA sequences to predict protein binding, histone modification, and chromatin accessibility (Alipanahi et al. 2015, Zhou and Troyanskaya 2015). Basset (Kelley et al. 2016) predicted the impact of non-coding variants on cell type specific DNase-seq profiles. With larger scale and finer resolution, Basenji (Kelley et al. 2018) incorporated distal interactions and predicted a much larger collection of epigenome profiles. ExPecto (Zhou et al. 2018) evaluated the tissue-specific gene expression changes for mutations. Cross-species investigations with deep neural networks (Kelley 2020, Minnoye et al. 2020) implicated a higher level regulatory code beyond strict sequence conservation as playing a significant role for predicting functionally similar non-coding regions. Minnoye et al. (2020) discovered examples of functionally similar enhancers that sequence-based methods failed to identify. Beyond sequence conservation, functional genomic annotations are important complementary information to determine functional similarity of non-coding regions between species (Kwon and Ernst 2021). Many studies have revealed the evolutionary landscape of genomes and epigenomes by comparing matched datasets across species (Odom et al. 2007, Brawand et al. 2011, Vierstra et al. 2014, Cheng et al. 2014, Villar et al. 2015, Gjoneska et al. 2015). EpiAlignment (Lu et al. 2019) is the first method that incorporates both matched ChIP-seq experiments and DNA sequences as the mapping units to identify orthologues between human and mouse. However, EpiAlignment allows for binary encoding of only one matched pair of functional genomic datasets, which provides limited information for discriminating a conserved epigenome against a random one.

**Figure 2:**
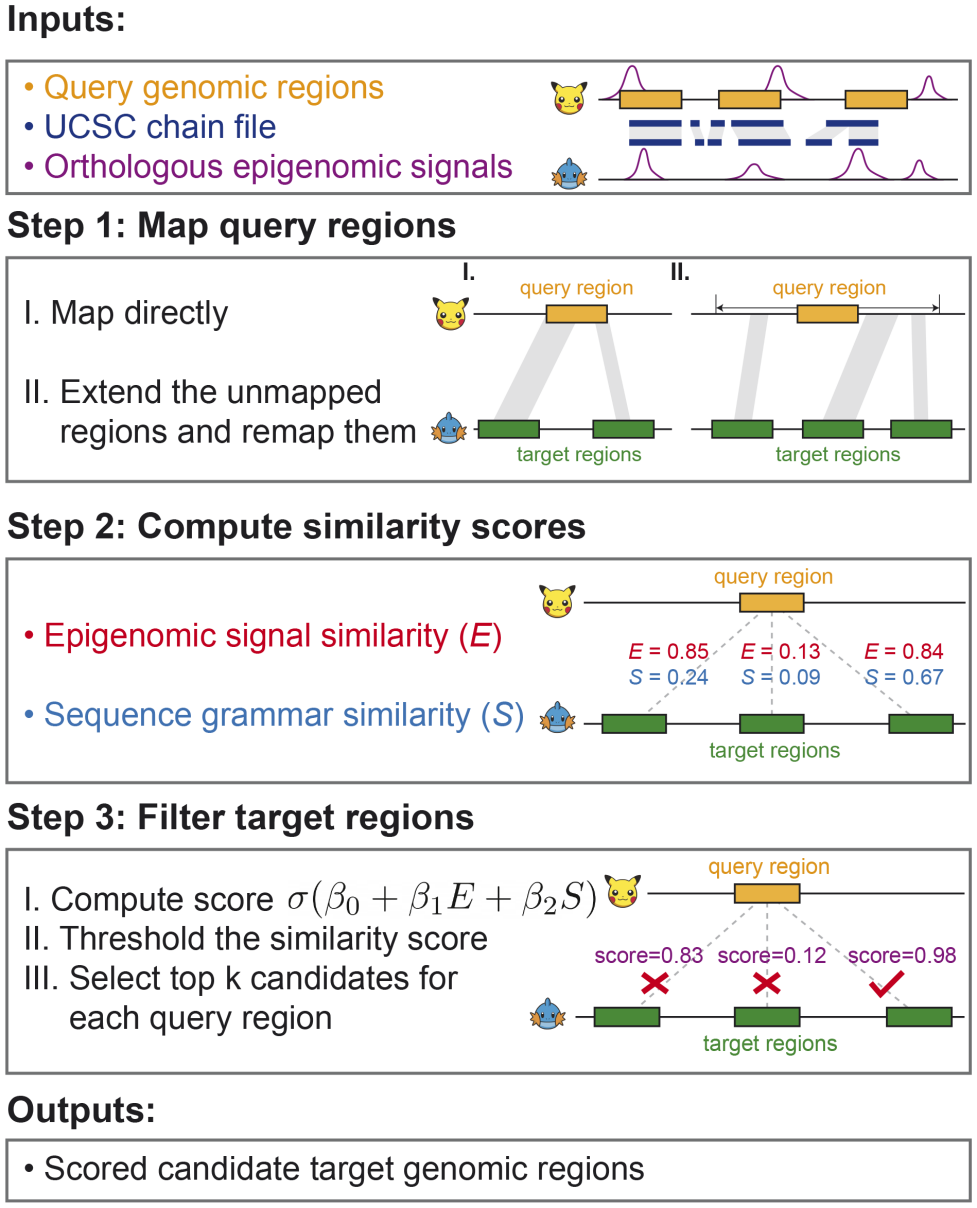
The AdaLiftOver workflow. The cartoon icons denote any two species with chain files. Top: query regions in the query genome; Bottom: target regions in the target genome. Inputs: genomic coordinates of the query regions, the UCSC chain file from the query genome to the target genome, and the matched epigenome datasets. Step 1: AdaLiftOver defaults to the UCSC liftOver if the query regions map successfully (I). If a query region does not map, AdaLiftOver extends the query region in a local window and applies the UCSC liftOver to this extended query region (II). AdaLiftOver merges small gaps among the resulting orthologous regions and generates candidate target regions based on these merged orthologous regions (indicated by translucent connection bands) with the same width as the query region. Step 2: AdaLiftOver uses local binary epigenomic and sequence grammar feature vectors to compute the similarity scores between the query region and each of the corresponding candidate target regions. Step 3: AdaLiftOver scores the candidate target regions with a logistic model ($\beta_{0}=-3,\beta_{1}=4,\beta_{2}=5$) based on their two similarity scores. The users can threshold these scores and rank the candidate target regions based on their probabilities of mapping to the query region. With score threshold of 0.4 or *k *=* *1, AdaLiftOver picks the rightmost candidate target region with estimated probability of mapping as 0.98. Outputs: For each query region, AdaLiftOver outputs a scored and filtered list of candidate target regions that are most similar to the query region in terms of regulatory information

To address the limitations of strictly sequence-based mapping of functionally similar non-coding regions and leverage higher-order regulatory grammar embedded in DNA sequences, we developed Adaptive liftOver (AdaLiftOver). AdaLiftOver is built on the UCSC liftOver framework for identifying and prioritizing orthologous regions by leveraging functional epigenome information. It enables mapping genomic coordinates between any two species with chain files and at least one pair of matched epigenome datasets. AdaLiftOver takes as input query genomic regions, the UCSC chain file, and one or more matched epigenome datasets (Fig. 2). We curated a list of matched epigenome datasets between human and mouse for general use from the ENCODE resources (Moore et al. 2020). AdaLiftOver allows the users to adaptively incorporate additional matched datasets and adjust the contribution of these datasets to the mapping. For each query region, AdaLiftOver generates a curated list of candidate target regions and prioritizes them with a score from a logistic model. The users can retrieve the final set of mapping regions by retaining only the top candidate target regions exceeding a score threshold (Fig. 2).

We applied AdaLiftOver to a variety of case studies including genomic intervals (peaks) from ATAC-seq and ChIP-seq experiments of seven different species including chicken, cow, horse, mouse, pig, rat, and zebrafish, and SNPs from GWAS datasets as queries. AdaLiftOver yields consistently superior performances than the competing methods for mapping of both the peaks across human and a variety of model organism genomes and the SNPs to the mouse genome. The R implementation for AdaLiftOver is available at https://github.com/keleslab/AdaLiftOver.

## 2 Materials and methods

### 2.1 AdaLiftOver framework

AdaLiftOver is a large-scale computational framework that leverages functional regulatory information to enhance the UCSC liftOver. Specifically, AdaLiftOver implements a two-step strategy for mapping each query genomic region *Q* (Fig. 2), which could constitute genomic intervals from biochemically active regions of the genome (i.e. ChIP-seq peaks) or GWAS SNPs. We first directly apply the UCSC liftOver to map *Q*. If *Q* fails to map directly, we extend *Q* with a local window on both sides and then apply the UCSC liftOver on the extended query region to generate candidate target regions. Let $O_{1},O_{2},\ldots ,O_{m}$ denote the resulting candidate regions. We note that it is possible to have no orthologous regions, i.e. *m *=* *0. For each orthologous region *O_j_*, j=1,…,m, AdaLiftOver generates a list of evenly spaced candidate target regions with a predefined resolution $T_{j,1},T_{j,2},\ldots ,T_{j,n_{j}}$, where $n_{j}\geq 1$ and their widths are set to be equal to that of *Q*. For simplicity, we denote all curated target genomic regions of *Q* as $T_{1},T_{2},\ldots ,T_{n}$, where $n=\sum_{j=1}^{m}n_{j}$. AdaLiftOver computes the local epigenomic feature vectors for *Q* and $T_{1},T_{2},\ldots ,T_{n}$ as *e_Q_* and $e_{T_{1}},e_{T_{2}},\ldots ,e_{T_{n}}$. Likewise, the local sequence grammar feature vectors are defined as *s_Q_* and, $s_{T_{1}},s_{T_{2}},\ldots ,s_{T_{n}}$. Then, the epigenomic and the sequence grammar feature similarities can be defined as $E_{i}=\text{sim}(e_{Q},e_{T_{i}})$ and $S_{i}=\text{sim}(s_{Q},s_{T_{i}})$, respectively, where i=1,2,…,n and sim(·) is a similarity function. AdaLiftOver scores each candidate target region *T_i_* with a logistic function $\sigma (\beta_{0}+\beta_{1}E_{i}+\beta_{2}S_{i})$, where $\sigma (x)=\frac{1}{1+e^{-x}}$ is the sigmoid function and $\left(\beta_{0},\beta_{1},\beta_{2}\right)$ are predefined logistic regression parameters estimable by the training module.

### 2.2 Regulatory information similarity

#### 2.2.1 Epigenomic features

We interrogated 67 matched ENCODE ChIP-seq and DNase-seq datasets between human and mouse from the following 10 tissues: heart, kidney, liver, lung, placenta, small intestine, spleen, stomach, testis, and thymus (Supplementary Section 1.2). These datasets are integrated into AdaLiftOver and the users can augment these with additional epigenome datasets from matched tissues and/or cell types. While there are a number of ways to summarize the signal from epigenome datasets, due to the computational challenges we articulated in Supplementary Section 1.4, we considered the local epigenomic features as 67-dimensional binary vectors from the overlap of the genomic regions with the peaks from the epigenome datasets. The choice of binarization provides a balance between the signal-to-noise ratio and the computational time, space, and memory requirements (Supplementary Section 1.4). We also remark that, in all the case studies that follow, the query samples are not from these 10 tissues used to derive the epigenome features to illustrate robustness of AdaLiftOver for mapping query regions of interest without directly relevant epigenome datasets.

#### 2.2.2 Sequence grammar features

We utilized 841 core vertebrate JASPAR motifs (Castro-Mondragon et al. 2022) as a list of ‘words’ capturing the high-level ‘grammar’ encoded by DNA sequences and are beyond traditional sequence alignment. We used ‘motifmatchr’ (Schep et al. 2017) for fast motif scanning in the vicinity of genomic regions instead of storing and querying the genome-wide motif occurrences. We define the sequence grammar feature of a query region as the 841-dimensional binary vector.

#### 2.2.3 Similarity metrics

For a pair of binary vectors $u,v\in R^{d}$, their weighted cosine similarity with weight $w\in R^{d}$ can be computed as:


$$\text{sim}(u,v)=\frac{\sum_{i=1}^{d}w_{i}u_{i}v_{i}}{\sqrt{\sum_{i=1}^{d}w_{i}u_{i}}\sqrt{\sum_{i=1}^{d}w_{i}v_{i}}}.$$

**Figure 3:**
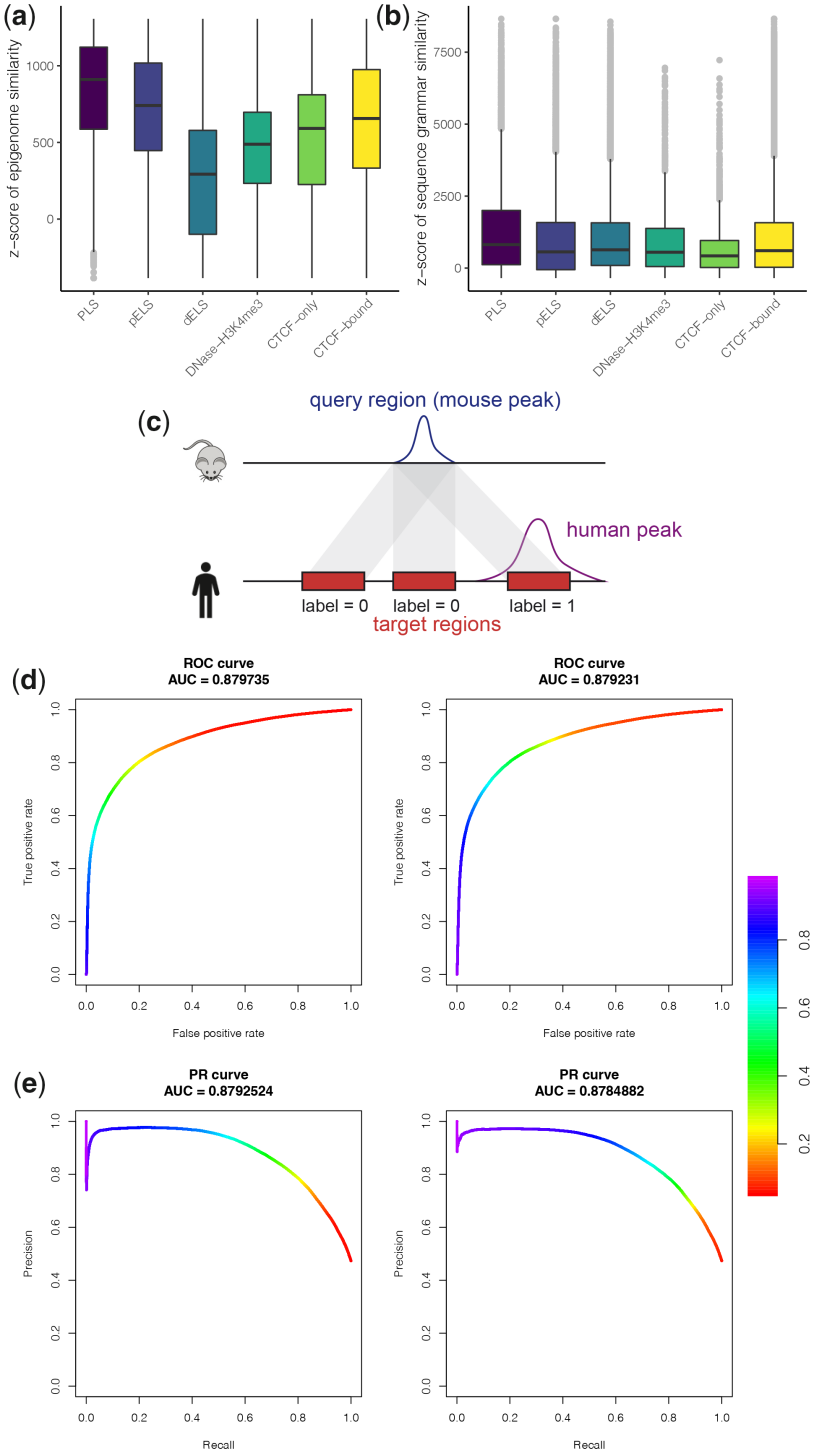
(a and b) Regulatory information similarity between orthologous cCREs. *X*-axis: six cCRE categories. A null distribution for each of the similarity scores is estimated by randomly permuting cCREs 10, 000 times. Observed similarity scores were transformed into *z*-scores using the mean and variance estimates of these null distributions. (a) The *z*-scores of the epigenomic feature similarity across the six cCRE categories. (b) The *z*-scores of the sequence grammar similarity across the 6 cCRE categories. (c) An illustration of labeling candidate target regions of a mouse query region with the corresponding human epigenome peaks. Positive and negative classes are represented by 1 and 0, respectively. The translucent connection bands represent candidate orthologous mappings. (d and e) The ROC and the PR curves with a local window size of 2 kb in the islet ATAC-seq study. Left panel: with the default parameters from the LOOCV experiments; Right panel: with the parameters from the refitted logistic regression. The color denotes the threshold for the logistic probability score

As default, AdaLiftOver uses equal weights while computing similarity scores. The users can specify the weights for computing the epigenomic feature similarities with different functional genomic datasets. This specific choice of the epigenomic and sequence grammar feature similarity metric is a result of our investigations on the ENCODE candidate *cis*-regulatory elements (cCREs) (Moore et al. 2020). We identified 103 529 orthologous cCREs between human and mouse using UCSC liftOver (Supplementary Section 1.3) and quantified their epigenomic and sequence grammar feature similarities as described above using the matched ENCODE epigenome datasets and motif scans of the JASPAR database. All the orthologous cCREs exhibited markedly higher epigenome similarities than randomly matched human and mouse cCREs, supporting a broad level of epigenome conservation between the orthologous regulatory elements (Fig. 3a). Moreover, promoter-like signatures, proximal enhancer-like signatures, and distal enhancer-like signatures exhibited monotonically decreasing epigenome conservation which further highlighted the affinity of the epigenomic feature similarity to capture different classes of regulatory elements. This decrease in the epigenomic feature similarity score going from promoters to distal enhancers can be attributed to the rapid divergence of enhancers compared to promoters during regulatory evolution (Cheng et al. 2014, Villar et al. 2015). Similarly, Fig. 3b illustrates that the sequence grammar similarity captures the degree of sequence conservation across all cCRE categories since UCSC liftOver-defined orthologues are based on pairwise sequence alignment results. We also observed that cosine similarity yielded better stability than the Jaccard similarity for binary features which further justified this choice (Supplementary Section 1.3).

#### 2.2.4 Parameter tuning with the training module

To tune the parameters of AdaLiftOver, we performed leave-one-out cross-validation (LOOCV) with the 67 matched ENCODE epigenome datasets. Specifically, for each fold of the cross-validation, we ensured that the datasets from the same tissue as the validation dataset were excluded from the training set (e.g. when mapping heart H3K4me3 peaks, all other epigenome data from heart were excluded from the epigenomic feature similarity calculations). We applied AdaLiftOver with a grid of window sizes from 0 to 5 kb and in increments of 400 bp. After mapping mouse query regions, i.e. peaks, we labeled the candidate target regions in the human genome as positives if they overlapped with the corresponding human epigenome peaks and as negatives otherwise (Fig. 3c). Further details on the generation of gold standard positive and negative orthologous pairs are provided in Supplementary Section 1.5. To learn the optimal weights of epigenomic and sequence grammar feature similarities, we fitted a logistic regression model with the two similarity features (Supplementary Section 1.5) and computed the area under receiver operating characteristic (ROC) and precision recall (PR) curves for this logistic fit. Optimizing the area under the ROC and PR curves yielded ∼2 kb as the optimal window size (AUROC: 0.820 ± 0.0113, AUPR: 0.604 ± 0.0371; Supplementary Section 1.5). We used 2 kb as the size of the local window in generating candidate target regions for the rest of this manuscript. In contrast to the stable local window size, the optimal logistic regression coefficients exhibited larger variability across different folds of the cross-validation. Therefore, we leveraged the averaged coefficient estimates as the weights for the two similarities in the logistic function (Supplementary Section 1.5). To facilitate training with other model organism data, we implemented an AdaLiftOver training module which allows users to estimate the logistic regression coefficients and experiment with thresholds for the logistic probability score.

To further investigate the robustness of the default parameters of AdaLiftOver set by the LOOCV experiments with the ENCODE repertoire, we applied AdaLiftOver on 46 676 mouse pancreatic islet ATAC-seq peaks (Dong et al. 2021) with widths between 150 bp and 3 kb as the query regions and performed the following experiment. After generating the candidate target regions at a grid of window sizes, we evaluated them by fitting the logistic regression with labeled data where the candidate target regions overlapping the gold standard human islet ATAC-seq peaks (Greenwald et al. 2019) were labeled as 1 and the rest as 0. First, we observed that the optimal window size of 2 kb from the LOOCV experiments agreed well with the optimal window size in this experiment (Supplementary Fig. S5). Next, we scored the candidate target regions generated at local window size of 2 kb with (1) the default parameters from the LOOCV experiments for the logistic regression and (2) parameters from the refitted logistic regression by labeling the candidate target regions as above. Overall, we observed that performance with parameters (#2 above) tuned on this experiment agreed well with the parameters (#1 above) trained with the LOOCV experiments of the 67 ENCODE epigenome datasets (Fig. 3d and e; AUROC: #1 above 0.880, #2 above 0.879; AUPR: #1 above 0.879, #2 above 0.878). This further justified the default parameter setting in AdaLiftOver. All the ROC and PR calculations were conducted with the R package PRROC (Grau et al. 2015).

#### 2.2.5 Enrichment analysis of mapped regions

To provide support for the mapped regions in the case studies we presented, we asked whether they resided within genomic regions with relevant epigenomic/genic features in the mapped genome more than expected by chance. The null distributions for quantifying these enrichments were adjusted for background genomic factors such as chromosomes and the PhyloP scores of the mapped regions (Supplementary Section 1.8).

## 3 Results

### 3.1 Large-scale evaluation of AdaLiftOver for generating candidate human epigenome datasets from model organism data

#### 3.1.1 Evaluation with large-scale TF ChIP-seq data from matched human and mouse cell lines

**Figure 4:**
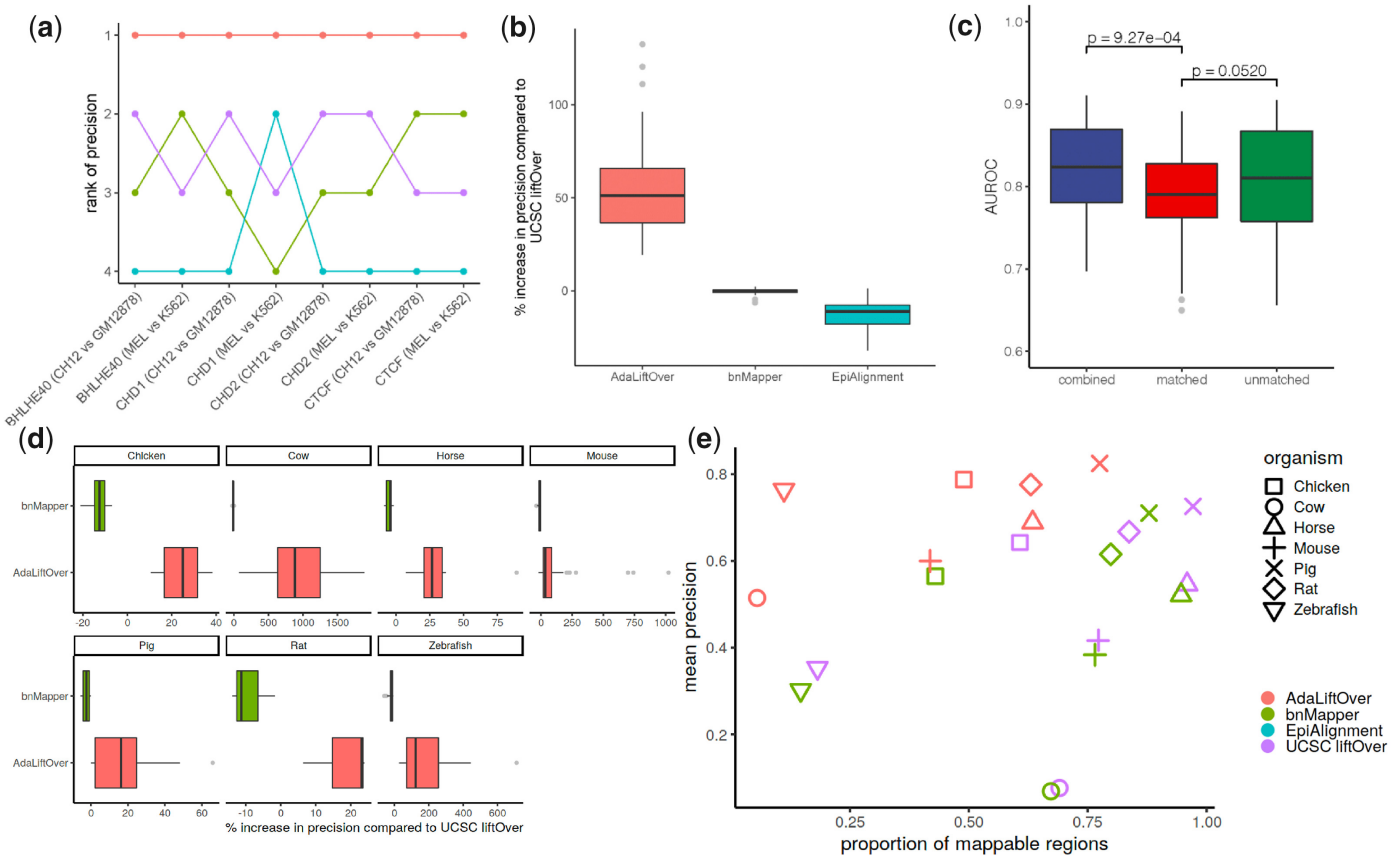
(a) Comparison of the ranks of the four orthologous mapping methods in terms of their precision over eight pairs of matched human-mouse TF ChIP-seq datasets that included results from EpiAlignment. (b) Percentage increase in precision of the methods compared to the state-of-the-art UCSC liftOver over 55 pairs of matched cell line human-mouse TF ChIP-seq datasets. (c) Comparison of performances of AdaLiftOver under three different configurations of epigenome dataset repertoire. Y-axis: area under the receiver operating characteristic curve. unmatched: model training by the default 67 pairs of ENCODE datasets excluding the relevant cell type; matched: model training by the open chromatin regions from the same cell type only; combined: model training with a weighted combination between the previous two where the relevant cell type receives 10× more weight. The *P*-values are computed from Mann–Whitney *U* tests. (d) Percentage increase in precision of AdaLiftOver and bnMapper compared to the state-of-the-art UCSC liftOver over 7 species. (e) Comparison of three orthologous mapping methods over 7 species. *Y*-axis: precision is defined as the (# of mapped regions with label 1)/(# of mapped regions). *X*-axis denotes the proportion of mappable query regions

We benchmarked AdaLiftOver against other orthologous mapping methods for a large collection of epigenome datasets from the ENCODE project. We utilized 55 human-mouse matched TF ChIP-seq peak sets (Cheng et al. 2014) from erythroid and lymphoblast cells (Supplementary Section 1.6). Specifically, we applied AdaLiftOver to 55 mouse ChIP-seq datasets with an average of 22 679 peaks. Due to the scalability issue of EpiAlignment, we applied EpiAlignment only on the 8 pairs of samples displayed in Fig. 4a. AdaLiftOver achieves the best precision while maintaining, on average, 4456 true positives compared to UCSC liftOver’s 4313 true positives (Fig. 4a and Supplementary Table S4) for all pairs of matched datasets. With an average precision of 0.372, AdaLiftOver boosts the precision by >50% compared to UCSC liftOver (Fig. 4b).

Mapping with AdaLiftOver in the above settings did not include any epigenome datasets from the tissues/cell types relevant to the query regions. Next, we asked whether including epigenomic feature from the relevant tissue/cell type impacted the performance. Specifically, we leveraged two pairs of matched ENCODE DNase-seq and ATAC-seq datasets from erythroid and lymphoblast cells (Supplementary Section 1.6). We observed that AdaLiftOver has a better performance with the default 67 out-of-sample epigenome datasets than using the relevant open chromation regions alone (Fig. 4c). This demonstrates the practical robustness of AdaLiftOver. As expected and revealed by Fig. 4c, combining all datasets (both the default repertoire and the epigenome dataset from the relevant tissue/cell type) yielded the best performance.

#### 3.1.2 Benchmarking across multiple species with matched epigenome datasets

Next, to go beyond human and mouse which are relatively closely related species, we mapped between human and six other species, namely, chicken (Kern et al. 2021), cow (Kern et al. 2021), horse (Kingsley et al. 2019), pig (Zhao et al. 2021), rat (Rintisch et al. 2014, Treviño et al. 2020, Lien et al. 2020), and zebrafish (Yang et al. 2020), and benchmarked AdaLiftOver in these settings. Details of number of tissues and the epigenome datasets utilized are provided in Supplementary Tables S6–S11 for the additional species and in Supplementary Tables S2 and S5 for mouse. Similar to the LOOCV experiments with the ENCODE human–mouse matched datasets in the Materials and Methods section, we performed LOOCV experiments (i.e. leave a single peak set out) with the matched datasets. In these experiments, we made sure to exclude the epigenomic data from the same tissue as the left-out data from the training set. EpiAlignment was excluded from this large-scale benchmarking study because of its lack of scalability.

Figure 4d summarizes the improvement in precision by AdaLiftOver compared to UCSC liftOver across all the LOOCV experiments and species (Supplementary Fig. S8a displays the average precision values). These results demonstrate that AdaLiftOver significantly improves precision when compared to liftOver, while bnMapper performs similarly or sometimes worse than liftOver across a wide range of model organisms. We observe that for some species such as zebrafish, AdaLiftOver achieves the best precision while maintaining, on average, 3994 true positives compared to UCSC liftOver’s 3238 true positives (Supplementary Table S6) for all pairs of matched datasets. In summary, with an average precision of 0.60–0.82 across the species, AdaLiftOver boosts the precision by an average of 17–951% compared to UCSC liftOver (Fig. 4d).

**Figure 5:**
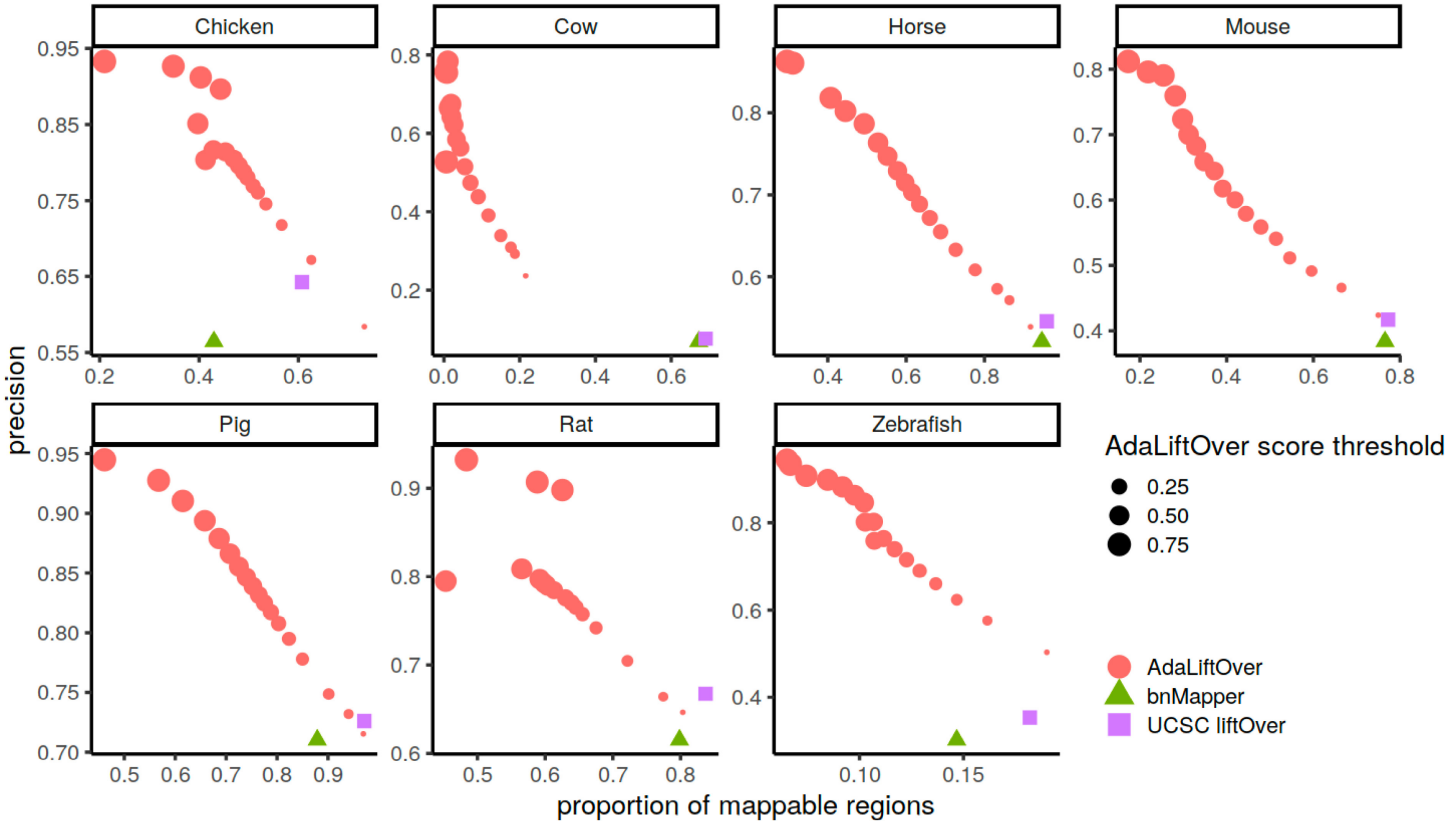
Precision versus proportion of mappable query regions for AdaLiftOver across seven species. These points are obtained by thresholding the logistic probability of mapping from AdaLiftOver. liftOver and bnMapper both generate single mapping results without a thresholdable parameter

A key advantage of AdaLiftOver is its ability to score and prioritize the regions that can be mapped to the model organism genome. Figure 4e displays precision as a function of proportion of mappable queries by each method (AdaLiftOver with default thresholds). We observe that for some species such as cow, AdaLiftOver identifies a smaller proportion of the query regions as mapping with high scores at a markedly higher precision compared to liftOver and bnMapper. We further investigated this by asking how the precision of AdaLiftOver varied as the logistic probability threshold is lowered to reach similar levels of proportion of mapped queries as liftOver. Figure 5 reveals that, as the logistic probability threshold is lowered, AdaLiftOver’s precision consistently stays higher than those of bnMapper and liftOver, highlighting its ability to prioritize the mapping regions. Furthermore, this analysis revealed that a threshold of 0.1 for the logistic probability of mapping yields increased precision compared to other methods, with minimal sacrifice in proportion of mappable queries. For species such as zebrafish and cow, using a smaller threshold of 0.05 results in >20% increase in precision without sacrificing the proportion. The results of this large-scale benchmarking are collectively further summarized in Supplementary Table S12.

Application of AdaLiftOver relies on the availability of matching epigenomic data in addition to the chain files of the species. We assessed the influence of the number of matching epigenomic datasets used in the training of AdaLiftOver. Utilizing the zebrafish ATAC-seq brain dataset as the query, we randomly sampled 3–27 samples from the zebrafish dataset collection, excluding those from the same tissue as the query data, and trained AdaLiftOver with these increasing numbers of datasets. We found that AdaLiftOver consistently outperformed bnMapper and UCSC liftOver, even with only three matching epigenome datasets in training. Furthermore, the precision steadily improved as the number of epigenome datasets increased (Supplementary Fig. S8b).

#### 3.1.3 AdaLiftOver prioritization of strictly sequence-based support between orthologous regions

We further utilized human and zebrafish brain ATAC-seq peak sets to explore to what extent regulatory information can overturn sequence-based support in mapping of orthologous regions. We considered two group of query regions in mapping of human brain ATAC-seq peaks to zebrafish:

Group I: query regions that map to target genome uniquely based on sequence conservation (e.g. with the UCSC liftOver).

Group II: query regions that map to multiple regions in the target genome (multi-mapper).

Then, we investigated the AdaLiftOver mapping scores of these regions. For regions in Group II, we looked at the differences between the maximum and minimum mapping scores after filtering scores <0.4 for all the regions (not to overcrowd the figure). Supplementary Fig. S9a summarizes the scores and illustrates the variation in the AdaLiftOver scores for these regions. First, for regions in Group I, we observe that 16.4% of them have mapping scores <0.5. This indicates that while these regions can be mapped strictly based on sequence-based conservation, the epigenomic profiles in the two species do not support regulatory conservation. This constitutes an example of overriding of sequence conservation. Second, when a query region can be mapped to multiple regions, these regions can achieve markedly different AdaLiftOver scores based on their regulatory grammar and epigenomic conservation (Supplementary Fig. S9b). This illustrates the ability of AdaLiftOver to resolve multiple mapping issues.

### 3.2 AdaLiftOver enables orthologous mapping for human GWAS SNPs

We considered three sets of GWAS SNPs to evaluate AdaLiftOver and the existing methods. The evaluations are largely based on evaluating whether the mapped regions were enriched in biologically relevant genomic regions (i.e. peaks from epigenome datasets of relevant cell types that were not utilized in mapping, neighborhood of GWAS phenotype-relevant genes) in the mapped genome.

#### 3.2.1 Case study I: Schizophrenia GWAS SNPs

**Figure 6:**
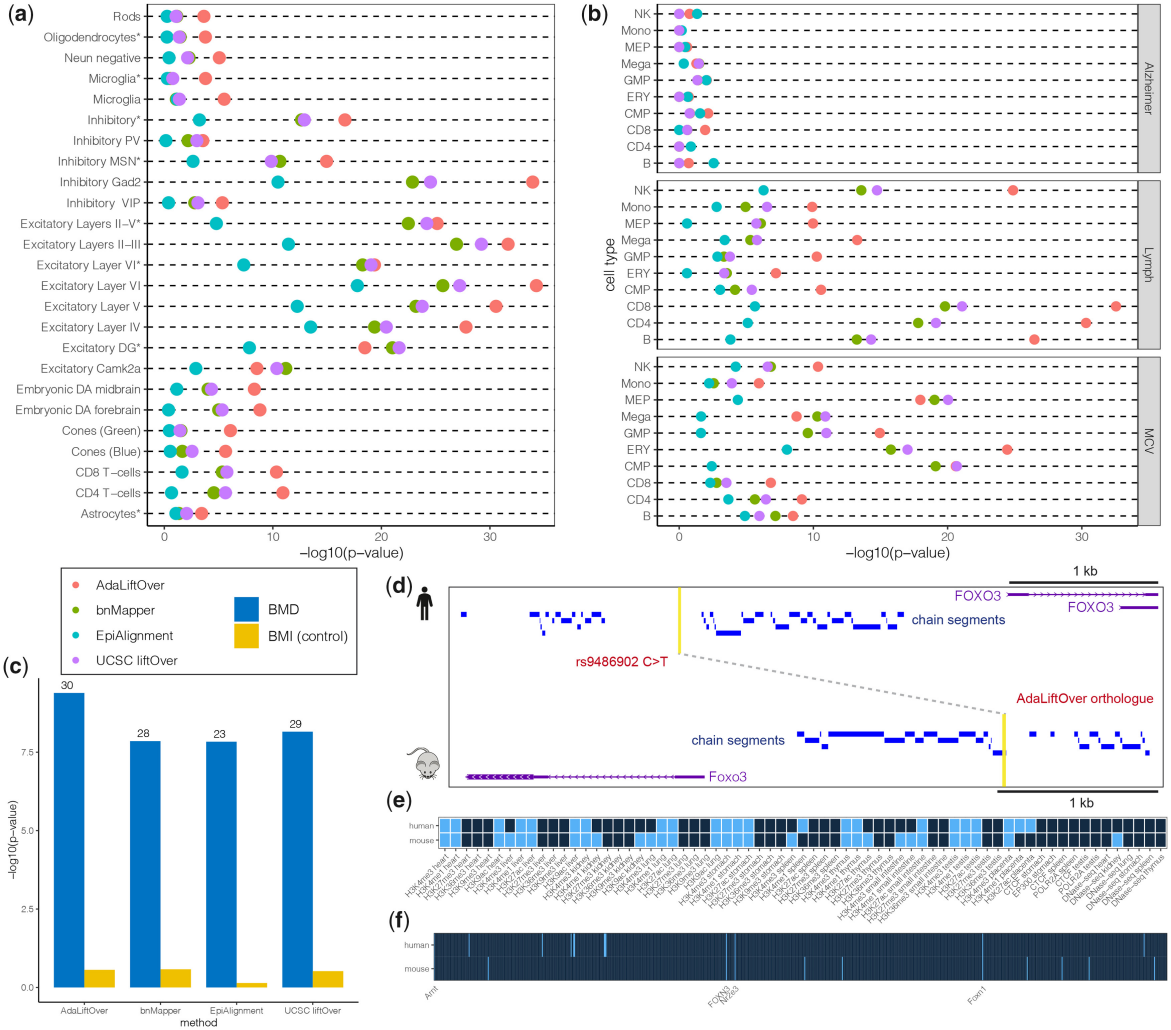
(a) Enrichment analysis for fine-mapped Schizophrenia GWAS SNPs with respect to ATAC-seq peaks from 25 cell populations. (b) Enrichment analysis for fine-mapped GWAS SNPs from three traits (Lymph and MCV are hematopoietic traits, Alzheimer is a control trait) with respect to 10 hematopoietic ATAC-seq peaks. Lymph: lymphocyte count; MCV: mean corpuscular volume. (c) Enrichment analysis for fine-mapped BMD GWAS SNPs with respect to 52 mouse BMD genes. BMI: body mass index (control trait). The numbers of BMD genes mapped are labeled on the top of each bar. (d–f) AdaLiftOver rescues and maps a BMD GWAS rs9486902 at the *FOXO3* promoter in human to *Foxo3* promoter in mouse. (d) The human GWAS and AdaLiftOver-derived mouse orthologue are highlighted and linked by a dashed line. The GWAS SNP rs9486902 fails to map using UCSC liftOver. (e and f) The binary epigenomic and sequence grammar feature profiles of the GWAS SNP and its AdaLiftOver orthologue. Light and dark shading denote 1 (overlap) and 0 (not overlap), respectively

To evaluate the performances of AdaLiftOver and other methods for mapping GWAS SNPs to model organism mouse, we investigated the 1648 fine-mapped Schizophrenia (SCZ) GWAS SNPs prioritized by Hook and McCallion (2020). We further utilized the large collection of mouse ATAC-seq data from 25 different brain cell populations out of 6 cell types (Hook and McCallion 2020) to evaluate the mapping results by their enrichment in the relevant cell populations. UCSC liftOver maps 715 (43.3%) GWAS SNPs where 1.47–19.1% of them overlap with each of the 25 mouse ATAC-seq datasets. In comparison, AdaLiftOver maps 612 (37.5%) GWAS SNPs and achieves a higher average precision of 8.07%. Figure 6a illustrates that AdaLiftOver displays a similar trend with stronger enrichment patterns than UCSC liftOver for the relevant cell populations. Consistent with the S-LDSC enrichment results by Hook and McCallion (2020), we find that SCZ GWAS SNPs are enriched in chromatin accessible regions of all the excitatory neurons and all the inhibitory neurons except PV and VIP (Supplementary Section 1.8). AdaLiftOver largely preserves the biological information of the SCZ GWAS SNPs after cross-species mapping.

#### 3.2.2 Case study II: hematopoiesis GWAS SNPs

To further evaluate AdaLiftOver and other methods in the GWAS SNPs setting, we leveraged human fine-mapped GWAS data for four hematopoietic traits: mean corpuscular volume (MCV), mean platelet volume (MPV), monocyte count (Mono), and lymphocyte count (Lymph) (Ulirsch et al. 2019). We mapped these SNPs to the mouse genome and performed enrichment analysis with the mouse ATAC-seq peaks from 10 blood cell types (Xiang et al. 2020). The enrichment analysis demonstrates that SNPs for MCV are enriched in chromatin accessible regions of ERY, MEP, CMP, and GMP cells; SNPs for Lymph are enriched for NK, CD4, CD8, and B cells (Fig. 6b, Supplementary Section 1.9). These observations are largely consistent with the g-chromVAR (Ulirsch et al. 2019) with the exception of MPV and Mono traits (Supplementary Section 1.9). For these set of mappings, AdaLiftOver and UCSC liftOver perform similarly in terms of enrichments of their mappings in relevant cell types.

#### 3.2.3 Case study III: bone mineral density GWAS SNPs

We next showcase how AdaLiftOver can be utilized to map UK Biobank SNPs (Sudlow et al. 2015) to mouse for further investigation. In a study of osteoporosis, Swan et al. (2020) reported 200 mouse genes as significantly altering bone mineral density (BMD) using BMD measures obtained from a large pool of mice genetically modified for deletion of individual genes. Swan et al. (2020) identified 52 human orthologues of these mouse BMD genes within a 250-kb distance range of UK Biobank BMD GWAS SNPs. To further leverage this knockout mouse resource, we mapped 3125 fine-mapped BMD GWAS SNPs (Morris et al. 2019, the UK Biobank with PIP ≥0.1) to the mouse genome and evaluated their enrichment for BMD genes. We used 3601 body mass index GWAS SNPs from the UK Biobank as negative controls. Compared to other methods, AdaLiftOver achieves the best enrichment results and is capable of identifying the most number of BMD genes (30/52) as relevant for human BMD GWAS SNPs (Fig. 6c and Supplementary Section 1.10). In order to associate more BMD genes with human GWAS SNPs, we then interrogated a larger set of 116 402 GWAS SNPs from the UK Biobank (PIP ≥0.001). As a result, AdaLiftOver maps to 90 BMD genes with 65.2% increase in precision compared to UCSC liftOver (Supplementary Section 1.10). Figure 6d–f illustrates an example where UCSC liftOver does not map any SNPs to the vicinity of the mouse BMD gene *Foxo3* gene but AdaLiftOver is able to rescue *Fox3* with mapping of a BMD GWAS SNP. The SNP rs9486902 resides at the promoter region of human gene *FOXO3* while it is located in a gap among human-mouse chain segments leading to a miss by UCSC liftOver. AdaLiftOver is able to identify a mouse orthologue at the promoter region of *Foxo3* that has similar epigenomic features (Fig. 6e) and sequence grammar (Fig. 6f). In particular, these human and mouse orthologous regions anchored by the *FOXO3* and *Foxo3* genes share common transcription factor binding site motifs that are relevant for BMD. Specifically, ARNT co-binds with Ahr which negatively influences osteoblast proliferation (Yu et al. 2014). FOXN3 interacts with Menin, the product of *MEN1*, which influences bone metabolism (Kaji 2012). Nr2e3, as a nuclear receptor (Oh et al. 2008), is related to human disorders including reduced BMD (Achermann and Jameson 2003, Achermann et al. 2017). Overall, 25.3% of these UK Biobank (PIP ≥0.001) GWAS SNPs can be mapped; however, the majority of them (92.7%) are mapped to ‘desert’ regions that are 250 kb away from the 200 BMD gene promoters, emphasizing the necessity for follow-up with 3D genome profiling assays such as pcHi-C (Mifsud et al. 2015) and its variants.

## 4 Discussion

We developed AdaLiftOver to enable mapping and prioritizing of non-coding regions between human and model organism genomes. AdaLiftOver takes as input UCSC chain files and matched epigenome datasets of the species to map query regions. It goes beyond traditional sequence alignment of comparative genomics for lifting over between genomes and simultaneously incorporates comparative epigenomics and sequence grammar similarity. To the best of our knowledge, this is the first systematic benchmark study of different orthologous mapping methods with comprehensive real biological data applications. Compared to other methods, AdaLiftOver is more accurate and robust, and offers a computationally inexpensive way of generating hard-to-obtain functional genomic datasets in other genomes by incorporating epigenomic and sequence grammar features. Table 1 further summarizes the flexibility, scalability, and running time of AdaLiftOver compared to existing methods.

**Table 1.** Technical comparison among existing orthologues mapping methods.

Method	Sequence alignment	Epigenomic features	Sequence grammar features	Scalability	Running time	Generalizability
AdaLiftOver	Yes	Multiple	Yes	Yes	∼20 min	Yes
bnMapper (Denas et al. 2015)	Yes	No	No	Yes	∼10 min	Yes
EpiAlignment (Lu et al. 2019)	Yes	One	No	No	∼3 h	No
UCSC liftOver (Hinrichs et al. 2006)	Yes	No	No	Yes	∼10 s	Yes

Sequence alignment: the dependence on the UCSC sequence alignment framework. Running time: the average running time for ∼20 000 TF ChIP-seq peaks. Generalizability: whether or not the method supports any genomes with chain files.

We found that the majority of orthologues of GWAS SNPs tend to have an enriched but low overlapping percentage with related open chromatin regions in mouse. We expect this result to improve as the epigenomic features leveraged span more cell types and dynamic conditions. In particular, developmental and disease trajectories revealed by single cell ATAC-seq might provide more enrichment for orthologues of GWAS SNPs. With a more comprehensive epigenome space, AdaLiftOver can serve as a versatile approach for pinpointing potential GWAS orthologues in a model organism and can facilitate high-throughput perturbation experiments. Currently, AdaLiftOver is restricted to binary features due to the space requirements and time complexity. We expect a more computationally efficient implementation of AdaLiftOver to incorporate features at other scales.

## Supplementary Material

btad149_Supplementary_Data
