# Supplementary material for: AdaLiftOver: high-resolution identification of orthologous regulatory elements with Adaptive liftOver
Source: Bioinformatics. 2023 Apr 2;39(4):btad149. doi: 10.1093/bioinformatics/btad149 (PMC10085516; doi:10.1093/bioinformatics/btad149)
Supplement: btad149_Supplementary_Data [file btad149_supplementary_data.pdf]

Supplementary Materials for “AdaLiftOver: High-resolution identification of orthologous regulatory elements with adaptive liftOver”

Chenyang Dong[1,a], Siqi Shen[2,a], and Sündüz Keleş[1,2,*]

[1]Department of Statistics,
[2]Department of Biostatistics and Medical Informatics,
[a]These authors contributed equally,
Corresponding author: [*]keles@stat.wisc.edu

Contents

1 Supplementary Notes **2**
1.1 The orthologous gene promoters . . . 2
1.2 The ENCODE datasets in the AdaLiftOver default repertoire for computing epigenomic feature similarities . . . 2
1.3 Evaluating similarity metrics with the ENCODE cCRE data . . . 2
1.4 Discussion on the choice of data formats and similarity metrics . . . 3
1.5 Parameter tuning for AdaLiftOver . . . 4
1.6 ENCODE and mouse ENCODE TF ChIP-seq datasets . . . 4
1.7 Benchmarking across multiple species with matched epigenome datasets . . . 5
1.8 Schizophrenia GWAS SNPs . . . 5
1.9 Hematopoiesis GWAS SNPs . . . 6
1.10 Bone Mineral Density GWAS SNPs . . . 6

2 Supplementary Tables **7**

3 Supplementary Figures **37**

1 Supplementary Notes

1.1 The orthologous gene promoters

We downloaded NCBI RefSeq gene annotation files from `https://hgdownload.soe.ucsc.edu/goldenPath/hg38/bigZips/genes/hg38.ncbiRefSeq.gtf.gz` and `https://hgdownload.soe.ucsc.edu/goldenPath/mm10/bigZips/genes/mm10.ncbiRefSeq.gtf.gz`. We utilized the human-mouse homology table from the Jackson Laboratory `http://www.informatics.jax.org/downloads/reports/HOM_MouseHumanSequence.rpt` and identified 16,374 orthologous genes in total. We defined the promoter region of a gene as 2 kb upstream and 200 bp downstream of the transcription start site (TSS). We explored two sets of chain files: the reciprocal chain files (”hg38.mm10.rbest.chain”, ”mm10.hg38.rbest.chain”) and the UCSC liftOver tool default chain files (”hg38ToMm10.over.chain.gz”, ”mm10ToHg38.over.chain.gz”) to determine the mappings between the orthologous promoters. We utilized both the UCSC liftOver command line tool by varying its various tuning parameters and its `R` implementation “rtracklayer::liftOver()” (a slim version without tuning parameters) to map all human/mouse promoters to the mouse/human genome and overlapped them with the corresponding counterparts. When a human/mouse promoter failed to map, we extended the promoters by 2 kb on both sides and remapped the extended region. In this context, we defined the positive and negative mappings as follows:

- True positive (TP): a promoter maps and overlaps with the correct counterpart.
- False positive (FP): a promoter maps but does not overlap with the correct counterpart.
- True negative (TN): a promoter fails to map or overlap with the correct counterpart after extending by 2 kb on both sides.
- False negative (FN): a promoter fails to map but it maps to the correct counterpart after extending by 2 kb on both sides.

The results are summarized in Supplementary Table 1.

1.2 The ENCODE datasets in the AdaLiftOver default repertoire for computing epigenomic feature similarities

The complete list of the datasets is provided in Supplementary Table 2.

1.3 Evaluating similarity metrics with the ENCODE cCRE data

The orthologous ENCODE cCRE data (v3) from both hg38 and mm10 were downloaded from `https://screen.encodeproject.org/`. We utilized the R function “rtracklayer::liftOver()” with the UCSC chain file “hg38.mm10.rbest.chain” to map human cCREs to mouse. The 103,529 orthologues were determined by requiring an overlap of at least 50% of the bases between the human cCRE orthologues and mouse cCREs. The human cCRE categories before and after mapping are summarized in Supplementary Figure 1.

With the 103,529 orthologous cCREs, we further evaluated the combinations of three different similarity metrics (Pearson correlation, cosine similarity, and soft cosine similarity[1]) and three different data formats summarizing the epigenomic signals in the form of peaks (BED format), fold change of normalized ChIP reads over control reads (Fold change BigWig), and

[1] soft_cosine$(u, v) = \frac{\sum_{i,j} s_{ij} u_i v_j}{\sqrt{\sum_{i,j} s_{ij} u_i u_j}\sqrt{\sum_{i,j} s_{ij} v_i v_j}}$, where s_{ij} was estimated by the sample Pearson correlation between the i-th and j-th entries of epigenomic feature vectors for all human and mouse cCREs.

p-values comparing ChIP and control reads along the genome (P-value BigWig). We specifically considered:

- BED format: we overlapped each cCRE with the list of peaks and quantified the strength of the signal as the percentage overlap with the peaks (numerical value between 0 and 1).
- Fold change BigWig format: we computed the arithmetic average of BigWig signals for each cCRE.
- P-value BigWig format: we computed the geometric average of BigWig signals for each cCRE.

Supplementary Figure 2 illustrates that the soft cosine similarity underperforms compared to the other metrics, and the fold-change BigWig format is outperformed by the p-value BigWig format as benchmarked by the z-scores. We observe that the BED format and the p-value BigWig format yield the best performance when combined with the Pearson correlation and the cosine similarity, respectively. Although the p-value BigWig format (with the Pearson correlation metric) achieves higher average z-scores compared to the BED format (with the cosine similarity metric), it exhibits larger variability across all 6 cCRE categories (Supplementary Figure 2). Therefore, the numerical features derived from the BED format data are as robust and informative as the BigWig formatted data.

The binary features constructed by overlapping the cCREs with the peaks (BED format data) attain similar performance as the numerical features derived from the BED format data (with the cosine similarity metric) (Supplementary Figure 3). A comparison of Supplementary Figures 2 and 3 indicates that the cosine similarity, which shows less variability than the Jaccard similarity, is a better similarity metric for binary vectors. We also incorporated the Jaccard similarity option in AdaLiftOver.

1.4 Discussion on the choice of data formats and similarity metrics

Epigenomic feature similarity. The 67 pairs of BigWig files from the ENCODE repertoire required $\sim$10 GB disk storage after being compressed from the $\sim$150 GB raw data to the Run-Length-Encoding structure separated by chromosomes. In contrast, only $<$50 MB space was required to store the binary signals of the BED files from the same ENCODE repertoire. Querying the BigWig format data, while it theoretically takes O(1) time complexity, was computational prohibitive for small servers and personal laptops. For a scientific computing server with Intel Xeon CPU E5-2680 v4 2.40GHz, it took $\sim$20 minutes to load the data in R and $\sim$24 hours to handle $\sim$100,000 orthologous genomic regions. However, querying the BED format data only took $\sim$1 minute for the same task. Further memory issues arose as the number of parallel jobs increase, rendering the overall approach using the BigWig numeric signals infeasible. On the contrary, the BED binary signals scaled well on a regular laptop. Therefore, AdaLiftOver leveraged binary features derived from the BED files with the cosine similarity metric for optimizing the disk storage, the computational speed, the scalability, and the performance robustness (Supplementary Figures 2 and 3).

Sequence grammar feature similarity. The pre-computed occurrence data for the 841 JASPAR motifs in the human and mouse genomes occupied $\sim$200 GB disk storage. Due to the high space requirement and to enable applicability to any model organism genome, we chose the motifmatchr R package, accelerated by C++ libraries, to perform on-demand motif scanning using PFM/PWMs. After quantifying motif occurrences, we used binary features and cosine similarity metric for the sequence grammar.

1.5 Parameter tuning for AdaLiftOver

Leave-one-out cross validation (LOOCV) with the ENCODE datasets. For each of the 67 ENCODE epigenome datasets in Supplementary Table 2, we utilized the remaining ENCODE datasets excluding those from the same tissue as the training data to compute epigenomic feature similarities. For a given set of human query regions (e.g., one of the human peak sets from Supplementary Table 2), we first identified candidate target regions in the mouse by lifting the query regions over to the mouse genome based on sequence conservation (R liftOver). Here, if a query region does not map, it gets extended on both sides and gets lifted over again. Then, these target regions in mouse are overlapped with the matched epigenomic data in the mouse and labeled as orthologous human-mouse pairs if they reside within a peak (Fig. 3c). This generates positive pairs: query region maps to mouse and the resulting target region overlaps with a peak in the matched peak set and negative pairs: query region maps to mouse but the resulting target region does not overlap with a peak in the matched peak set. These sets of positive and negative pairs are utilized for true/false positive calls. Supplementary Figure 4 illustrates that the optimal window size lies between 1 kb and 3 kb when evaluated across a a grid of window sizes by considering the trade-off between AUPR and AUROC. In practice, we suggest using a window size within this 1 to 3 kb range.

At window size of 2 kb, we summarize below the estimated logistic regression coefficients (mean $\pm$ s.e.; $\hat{\beta}_0, \hat{\beta}_1, \hat{\beta}_2$ denote the estimated coefficients for the intercept, epigenomic feature similarity, and sequence grammar feature similarity, respectively):

- $\hat{\beta}_0 = -4.16 \pm 0.231$
- $\hat{\beta}_1 = 5.40 \pm 0.349$
- $\hat{\beta}_2 = 2.06 \pm 0.212$

We used this set of parameters as default for the rest of the manuscript for mapping between human and mouse. With the constraint of filtering at most 50% of candidate target regions, we identified logistic probability score thresholds 0.376$\pm$0.0374 that maximize the precision 0.554$\pm$0.0371 using the AdaLiftOver training module. We selected the score threshold = 0.4 for the case studies on ATAC-seq and ChIP-seq peaks. The effects of selecting a logistic probability score threshold can be visually investigated with ROC/PR curves. The users may increase/decrease the score threshold if more stringent/lenient results are needed. We also considered an interaction term between the epigenomic feature similarity and the sequence grammar feature similarity in the logistic regression. However, the area under the curves did not yield significant differences with or without an interaction term (Mann-Whitney test p-values: 0.9752 for AUROC, 0.9539 for AUPR). AdaLiftOver implementation allows the inclusion of such an interaction term which might impact the results in other datasets.

1.6 ENCODE and mouse ENCODE TF ChIP-seq datasets

We utilized 55 pairs of TF ChIP-seq datasets from Cheng *et al.*, 2014 (Supplementary Table 3). The methods were run under the following settings:

- AdaLiftOver: threshold = 0.4. The chain file is "mm10.hg38.rbest.chain"
- bnMapper: Default parameters. The chain file is "mm10.hg38.rbest.chain"
- UCSC liftOver: The R function "rtracklayer::liftOver()". The chain file is "mm10ToHg38.over.chain"

- EpiAlignment: window = 2 kb. To tune EpiAlignment fairly, for MEL vs K562 samples, we used paired Erythrold cells H3K4me3 ChIP-seq data as the epigenome data input; For CH12 vs GM12878 samples, we used Lymphoblast cells H3K4me3 ChIP-seq data as the epigenome data input.

The results are summarized in in Supplementary Table 4.

We then downloaded the following ENCODE ATAC-seq/DNase-seq datasets to evaluate the performances of AdaLiftOver with matched tissues/cell types:

- mouse-CH12, DNase-seq, ENCFF855RCO.bed
- human-GM12878, ATAC-seq, ENCFF470YYO.bed
- mouse-MEL, DNase-seq, ENCFF726EZS.bed
- human-K562, ATAC-seq, ENCFF558BLC.bed

When combining the above epigenome profiles with the default ENCODE epigenome repertoire, we set the weights (w) of the epigenomic features from the matched tissues by a factor of 10 more in the similarity calculations.

1.7 Benchmarking across multiple species with matched epigenome datasets

We included six additional species, namely chicken, cow (7 and 8 samples, respectively, from Kern *et al.*, 2021), horse (8 samples from Kingsley *et al.*, 2019), pig (15 samples from Zhao *et al.*, 2021), rat (3 samples from Rintisch *et al.*, 2014; Treviño *et al.*, 2020; Lien *et al.*, 2020) and zebrafish (30 samples from Yang *et al.*, 2020) to our comparisons. This brought the total number of species to 7.

We used the corresponding rbest chain files for each of these species. We used a leave-one-out cross-validation strategy similar to that of Subsection 1.6 for the human-mouse experiments of matched epigenomic datasets, with a probability score threshold of 0.4.

Furthermore, we also added new modules for the additional species to the AdaLiftOver `R` package vignette, where we similarly used averages of the logistic regression parameter estimates from folds of the cross-validation experiments.

1.8 Schizophrenia GWAS SNPs

We utilized 1,648 SCZ GWAS SNPs (annotation PIP $\geq$ 0.1) and 25 mouse ATAC-seq datasets from Hook and McCallion, 2020. The benchmarking results are summarized in Supplementary Table 13.

The methods were run under the following settings:

- AdaLiftOver: threshold = 0.2. The chain file is “hg19.mm10.rbest.chain”
- bnMapper: Default parameters. The chain file is “hg19.mm10.rbest.chain”
- UCSC liftOver: The R function “rtracklayer::liftOver()”. The chain file is “hg19ToMm10.over.chain”
- EpiAlignment: window = 2 kb. GWAS SNPs from hg19 were converted to hg38 with UCSC liftOver using the chain file “hg19ToHg38.over.chain”. Due to the lack of BMD related orthologous epigenome data, we used orthologous cCRE data as the user inputted epigenomic signals in order to tune EpiAlignment fairly.

To provide support for the mapped GWAS SNPs, we asked whether ATAC-seq peaks of relevant cell types overlapped with the mapped GWAS SNPs more than expected by chance. Our calculations took into account background genomic characteristics such as sequence conservation as quantified by the PhyloP scores[2] and genomic coordinates as quantified by the chromosomes that the ATAC-seq peaks reside in. We first binned all the open chromatin regions[3] of the genome into intervals of 100 bp and stratified these with respect to their PhyloP scores discretized at 0.1 resolution for each chromosome separately. Then, we computed the empirical probability of overlap (p_i) with the mapped GWAS SNPs within each strata S_i, $i = 1 \cdots, I$, where index i runs over the PhyloP score grid and the numbers of chromosomes. Next, for a given ATAC-seq peak set of size P, we first calculated the expected proportion of overlap with the GWAS SNPs as $p_0 = \frac{1}{P}\sum_p \sum_i I(p\text{-th peak is in strata } i)p_i$. Using this null proportion, we evaluated the enrichment with a Binomial test that assessed whether the observed overlap of the ATAC-seq peak set with the mapped GWAS SNPs is larger than that can be explained by this expected proportion of overlap.

1.9 Hematopoiesis GWAS SNPs

We leveraged 10 blood-related mouse ATAC-seq peaks (Xiang *et al.*, 2020) and fine-mapped hematopoietic GWAS SNPs (Ulirsch *et al.*, 2019) with PIP $\geq$ 0.1 from 4 blood traits: MCV, MPV, Mono, Lymph. The settings of the methods compared and the enrichment analysis were the same as the SCZ GWAS case. The benchmarking results are summarized in Supplementary Table 14. The enrichment results for Mono (monocyte count) and MPV (mean platelet volume) are displayed in Supplementary Figure 10.

1.10 Bone Mineral Density GWAS SNPs

The promoter regions of BMD genes were defined as 2 kb upstream and 200 bp downstream of TSS. We utilized 1,097 fine-mapped BMD GWAS SNPs from Morris *et al.*, 2019 as well as 2,088 estimated BMD fine-mapped GWAS SNPs from the UK Biobank (SuSIE PIP $\geq$ 0.1). For negative controls, we utilized a comparable number of 3,601 BMI SNPs from the UK Biobank (SuSIE PIP $\geq$ 0.05). The settings of the methods compared and the enrichment analysis were the same as the SCZ GWAS case. The regions for enrichment analysis were the 52 BMD gene promoters and the constructed universe consisted of all orthologous gene promoters identified in Section 1.1. The results are summarized in Supplementary Table 15.

We further used 116,402 fine-mapped GWAS SNPs from the UK Biobank (SuSIE PIP $\geq$ 0.001). We didn't run EpiAlignment due to the lack of scalability of this method. The results are summarized in Supplementary Table 16.

[2]The PhyloP dataset was downloaded from `http://hgdownload.cse.ucsc.edu/goldenpath/mm10/phyloP60way/mm10.60way.phyloP60way.bw`

[3]The 100bp-binned open chromatin regions as the union of all target ATAC-seq peaks extended by 1 kb.

2 Supplementary Tables

Panel A: R liftOver (`rtracklayer::liftOver`) with Over chain file

	TP	FP	TN	FN
**human to mouse**	12,771	2,842	556	205
**mouse to human**	12,979	2,703	531	161

Panel B: R liftOver (`rtracklayer::liftOver`) with rbest chain file

	TP	FP	TN	FN
**human to mouse**	12,760	2,813	592	209
**mouse to human**	12,760	2,525	933	156

Panel C: UCSC liftOver with minMatch=0.1, and Over chain file

	TP	FP	TN	FN
**human to mouse**	12,431.52	2,758.24	776.56	407.68
**mouse to human**	12,767.60	2,540.56	644.64	421.20

Panel D: UCSC liftOver with minMatch=0.1, and rbest chain file

	TP	FP	TN	FN
**human to mouse**	12,380.64	2,681.72	886.76	424.88
**mouse to human**	12,519.80	2,361.48	1,068.56	424.16

Supplementary Table 1: Mapping of 16,374 orthologous gene promoters between human and mouse with R implementation of UCSC liftOver via `rtracklayer::liftOver` and UCSC liftOver command line tool under varying set of parameters and with two different chain files: Over and rbest. Parameter `minMatch` represents the minimum ratio of bases that must map for a successful liftOver (default of 0.1 as the most commonly used value for mapping between species). For panels C and D, the results are averaged over combinations of minChainT and minChainQ parameters that represent the minimum chain size in target and query genomes, respectively. These parameters were varied as: minChainT $\in \{0, 50, 100, 150, 200\}$ and minChainQ $\in \{0, 50, 100, 150, 200\}$. Overall results were insensitive to the combinations of these two parameters (data not shown), hence, prompting averaging of the results across these combinations of these two parameters. The calculations of TP, FP, TN, and FN are carried out as described in Supplementary Materials Section 1.1.

	Assay	Target	Tissue	mouse file id	human file id
1	ChIP-seq	H3K4me3	heart	ENCFF925JLJ.bed	ENCFF685WYG.bed
2	ChIP-seq	H3K4me1	heart	ENCFF577SAP.bed	ENCFF109AVD.bed
3	ChIP-seq	H3K27me3	heart	ENCFF337GMV.bed	ENCFF594SIJ.bed
4	ChIP-seq	H3K36me3	heart	ENCFF412PEF.bed	ENCFF501WXA.bed
5	ChIP-seq	H3K9me3	heart	ENCFF018ZTP.bed	ENCFF472HHB.bed
6	ChIP-seq	H3K9ac	heart	ENCFF043BXJ.bed	ENCFF305FQY.bed
7	ChIP-seq	H3K4me3	liver	ENCFF233NKJ.bed	ENCFF178DYP.bed
8	ChIP-seq	H3K4me1	liver	ENCFF626UMI.bed	ENCFF872NIB.bed
9	ChIP-seq	H3K27ac	liver	ENCFF734MGU.bed	ENCFF805YRQ.bed
10	ChIP-seq	H3K27me3	liver	ENCFF290NCY.bed	ENCFF977LFS.bed
11	ChIP-seq	H3K36me3	liver	ENCFF958ZWB.bed	ENCFF050ODT.bed
12	ChIP-seq	H3K9me3	liver	ENCFF763XZB.bed	ENCFF093NPF.bed
13	ChIP-seq	H3K9ac	liver	ENCFF025NBX.bed	ENCFF766JJU.bed
14	ChIP-seq	H3K4me3	kidney	ENCFF870HWW.bed	ENCFF917SIV.bed
15	ChIP-seq	H3K4me1	kidney	ENCFF661JHH.bed	ENCFF224BUW.bed
16	ChIP-seq	H3K27me3	kidney	ENCFF357TDA.bed	ENCFF775YUI.bed
17	ChIP-seq	H3K36me3	kidney	ENCFF219HDC.bed	ENCFF839LUB.bed
18	ChIP-seq	H3K9me3	kidney	ENCFF616DMW.bed	ENCFF163PII.bed
19	ChIP-seq	H3K9ac	kidney	ENCFF226CKB.bed	ENCFF138PYK.bed
20	ChIP-seq	H3K4me3	lung	ENCFF142XFR.bed	ENCFF254OZF.bed
21	ChIP-seq	H3K4me1	lung	ENCFF262JML.bed	ENCFF908EUN.bed
22	ChIP-seq	H3K27ac	lung	ENCFF455ADY.bed	ENCFF314QTE.bed
23	ChIP-seq	H3K27me3	lung	ENCFF536OYS.bed	ENCFF112CDW.bed
24	ChIP-seq	H3K36me3	lung	ENCFF262QMM.bed	ENCFF089HKW.bed
25	ChIP-seq	H3K9me3	lung	ENCFF737BJL.bed	ENCFF284QKU.bed
26	ChIP-seq	H3K9ac	lung	ENCFF871DGI.bed	ENCFF330TSX.bed
27	ChIP-seq	H3K4me3	stomach	ENCFF902SVE.bed	ENCFF494BWU.bed
28	ChIP-seq	H3K4me1	stomach	ENCFF377USJ.bed	ENCFF475EJU.bed
29	ChIP-seq	H3K27ac	stomach	ENCFF643VAF.bed	ENCFF978AOD.bed
30	ChIP-seq	H3K27me3	stomach	ENCFF993YFO.bed	ENCFF692HXY.bed
31	ChIP-seq	H3K36me3	stomach	ENCFF704KRU.bed	ENCFF243EAW.bed
32	ChIP-seq	H3K9me3	stomach	ENCFF401WHJ.bed	ENCFF299EII.bed
33	ChIP-seq	H3K4me3	spleen	ENCFF040ATH.bed	ENCFF636LHJ.bed
34	ChIP-seq	H3K4me1	spleen	ENCFF332TVZ.bed	ENCFF997KKX.bed
35	ChIP-seq	H3K27ac	spleen	ENCFF675SAZ.bed	ENCFF805FFP.bed
36	ChIP-seq	H3K27me3	spleen	ENCFF472CRY.bed	ENCFF867NGE.bed
37	ChIP-seq	H3K36me3	spleen	ENCFF962GUW.bed	ENCFF593PUF.bed
38	ChIP-seq	H3K4me3	thymus	ENCFF462HWJ.bed	ENCFF963LSO.bed
39	ChIP-seq	H3K4me1	thymus	ENCFF149ZSO.bed	ENCFF821ZRD.bed
40	ChIP-seq	H3K27ac	thymus	ENCFF476TDY.bed	ENCFF668BZJ.bed
41	ChIP-seq	H3K27me3	thymus	ENCFF497BVT.bed	ENCFF641CCT.bed
42	ChIP-seq	H3K36me3	thymus	ENCFF394EHO.bed	ENCFF869FKJ.bed
43	ChIP-seq	H3K4me3	small intestine	ENCFF250OKJ.bed	ENCFF638SMB.bed
44	ChIP-seq	H3K4me1	small intestine	ENCFF061UOC.bed	ENCFF377UAM.bed
45	ChIP-seq	H3K27ac	small intestine	ENCFF371CEP.bed	ENCFF074WDV.bed
46	ChIP-seq	H3K27me3	small intestine	ENCFF359SCM.bed	ENCFF644QDF.bed
47	ChIP-seq	H3K36me3	small intestine	ENCFF841SYQ.bed	ENCFF694AKL.bed
48	ChIP-seq	H3K4me3	testis	ENCFF582PBS.bed	ENCFF244NRV.bed
49	ChIP-seq	H3K4me1	testis	ENCFF868HGX.bed	ENCFF600MJO.bed

Continued on next page

Supplementary Table 2 – continued from previous page

	Assay	Target	Tissue	mouse file id	human file id
50	ChIP-seq	H3K27ac	testis	ENCFF047JTI.bed	ENCFF540YUQ.bed
51	ChIP-seq	H3K27me3	testis	ENCFF031KAC.bed	ENCFF319CMK.bed
52	ChIP-seq	H3K36me3	testis	ENCFF187TKX.bed	ENCFF469ASG.bed
53	ChIP-seq	H3K4me3	placenta	ENCFF296GDX.bed	ENCFF829GCX.bed
54	ChIP-seq	H3K4me1	placenta	ENCFF499ASE.bed	ENCFF867CRG.bed
55	ChIP-seq	H3K27ac	placenta	ENCFF226XOZ.bed	ENCFF297NSU.bed
56	ChIP-seq	CTCF	stomach	ENCFF907XEG.bed	ENCFF017NAF.bed
57	ChIP-seq	EP300	stomach	ENCFF132DDY.bed	ENCFF099PQM.bed
58	ChIP-seq	CTCF	spleen	ENCFF089EWX.bed	ENCFF645YCX.bed
59	ChIP-seq	POLR2A	spleen	ENCFF313QNI.bed	ENCFF683YQZ.bed
60	ChIP-seq	CTCF	testis	ENCFF033UQX.bed	ENCFF917CYG.bed
61	ChIP-seq	POLR2A	testis	ENCFF789JUX.bed	ENCFF967TFR.bed
62	DNase-seq		heart	ENCFF846XYQ.bed	ENCFF861CQD.bed
63	DNase-seq		kidney	ENCFF890GCF.bed	ENCFF388NNQ.bed
64	DNase-seq		lung	ENCFF459KBR.bed	ENCFF691GQY.bed
65	DNase-seq		stomach	ENCFF220GXC.bed	ENCFF731YGM.bed
66	DNase-seq		spleen	ENCFF343KXE.bed	ENCFF523NCT.bed
67	DNase-seq		thymus	ENCFF266KGB.bed	ENCFF494PNZ.bed

Supplementary Table 2: The 67 matched ENCODE datasets between mouse and human. These datasets make up AdaLiftOver's default epigenome repertoire.

	TF	human-K562	mouse-MEL	human-GM12878	mouse-CH12
1	BHLHE40	Yes	Yes	Yes	Yes
2	CHD1	Yes	Yes	Yes	Yes
3	CHD2	Yes	Yes	Yes	Yes
4	CTCF	Yes	Yes	Yes	Yes
5	E2F4	Yes	Yes	Yes	Yes
6	ELF1	Yes	Yes	Yes	Yes
7	EP300	Yes	Yes	Yes	Yes
8	ETS1	Yes	Yes	Yes	Yes
9	GATA1	Yes	Yes	No	No
10	JUND	Yes	Yes	Yes	Yes
11	MAFK	Yes	Yes	No	No
12	MAX	Yes	Yes	Yes	Yes
13	MAZ	Yes	Yes	Yes	Yes
14	MEF2A	Yes	Yes	Yes	Yes
15	MXI1	Yes	Yes	Yes	Yes
16	MYC	Yes	Yes	Yes	Yes
17	NRF1	Yes	Yes	Yes	Yes
18	POLR2A	Yes	Yes	Yes	Yes
19	RAD21	Yes	Yes	Yes	Yes
20	RCOR1	Yes	Yes	Yes	Yes
21	RDBP	Yes	Yes	No	No
22	SIN3A	Yes	Yes	Yes	Yes
23	SMC3	Yes	Yes	Yes	Yes
24	TAL1	Yes	Yes	No	No
25	TBP	Yes	Yes	Yes	Yes
26	UBTF	Yes	Yes	No	No
27	USF1	Yes	Yes	Yes	Yes
28	USF2	Yes	Yes	Yes	Yes
29	IRF4	No	No	Yes	Yes
30	KAT2A	No	No	Yes	Yes
31	PAX5	No	No	Yes	Yes
32	TCF12	No	No	Yes	Yes

Supplementary Table 3: Matched human and mouse TF ChIP-seq datasets used for benchmarking.

	# peaks	# map	# overlap	precision	sample	method
1	43498	18834	6761	0.36	BHLHE40 (CH12 vs GM12878)	AdaLiftOver
2	43498	32606	5970	0.18	BHLHE40 (CH12 vs GM12878)	bnMapper
3	43498	37751	6059	0.16	BHLHE40 (CH12 vs GM12878)	EpiAlignment
4	43498	33589	6152	0.18	BHLHE40 (CH12 vs GM12878)	UCSC liftOver
5	16325	8080	2911	0.36	BHLHE40 (MEL vs K562)	AdaLiftOver
6	16325	11716	2450	0.21	BHLHE40 (MEL vs K562)	bnMapper
7	16325	13803	2471	0.18	BHLHE40 (MEL vs K562)	EpiAlignment
8	16325	12076	2518	0.21	BHLHE40 (MEL vs K562)	UCSC liftOver
9	11286	6984	1417	0.20	CHD1 (CH12 vs GM12878)	AdaLiftOver
10	11286	9209	1049	0.11	CHD1 (CH12 vs GM12878)	bnMapper
11	11286	10071	1118	0.11	CHD1 (CH12 vs GM12878)	EpiAlignment
12	11286	9640	1114	0.12	CHD1 (CH12 vs GM12878)	UCSC liftOver
13	8170	6103	2269	0.37	CHD1 (MEL vs K562)	AdaLiftOver
14	8170	6686	1566	0.23	CHD1 (MEL vs K562)	bnMapper
15	8170	7336	1774	0.24	CHD1 (MEL vs K562)	EpiAlignment
16	8170	7036	1680	0.24	CHD1 (MEL vs K562)	UCSC liftOver
17	19553	11302	5176	0.46	CHD2 (CH12 vs GM12878)	AdaLiftOver
18	19553	15579	4589	0.29	CHD2 (CH12 vs GM12878)	bnMapper
19	19553	17297	4633	0.27	CHD2 (CH12 vs GM12878)	EpiAlignment
20	19553	16135	4787	0.30	CHD2 (CH12 vs GM12878)	UCSC liftOver
21	5062	3497	1727	0.49	CHD2 (MEL vs K562)	AdaLiftOver
22	5062	4009	1542	0.38	CHD2 (MEL vs K562)	bnMapper
23	5062	4428	1553	0.35	CHD2 (MEL vs K562)	EpiAlignment
24	5062	4194	1615	0.39	CHD2 (MEL vs K562)	UCSC liftOver
25	60828	22812	13086	0.57	CTCF (CH12 vs GM12878)	AdaLiftOver
26	60828	36477	14918	0.41	CTCF (CH12 vs GM12878)	bnMapper
27	60828	48665	13944	0.29	CTCF (CH12 vs GM12878)	EpiAlignment
28	60828	37944	15395	0.41	CTCF (CH12 vs GM12878)	UCSC liftOver
29	46608	18792	12015	0.64	CTCF (MEL vs K562)	AdaLiftOver
30	46608	26427	13862	0.52	CTCF (MEL vs K562)	bnMapper
31	46608	36707	12972	0.35	CTCF (MEL vs K562)	EpiAlignment
32	46608	27488	14299	0.52	CTCF (MEL vs K562)	UCSC liftOver
33	5579	3863	1358	0.35	E2F4 (CH12 vs GM12878)	AdaLiftOver
34	5579	4477	1140	0.25	E2F4 (CH12 vs GM12878)	bnMapper
35	5579	4693	1207	0.26	E2F4 (CH12 vs GM12878)	UCSC liftOver
36	4122	3319	1813	0.55	E2F4 (MEL vs K562)	AdaLiftOver
37	4122	3362	1509	0.45	E2F4 (MEL vs K562)	bnMapper
38	4122	3540	1590	0.45	E2F4 (MEL vs K562)	UCSC liftOver
39	26680	16032	9681	0.60	ELF1 (CH12 vs GM12878)	AdaLiftOver
40	26680	20849	8384	0.40	ELF1 (CH12 vs GM12878)	bnMapper
41	26680	21699	8775	0.40	ELF1 (CH12 vs GM12878)	UCSC liftOver
42	18949	12227	7511	0.61	ELF1 (MEL vs K562)	AdaLiftOver
43	18949	14536	6310	0.43	ELF1 (MEL vs K562)	bnMapper
44	18949	15086	6568	0.44	ELF1 (MEL vs K562)	UCSC liftOver
45	33645	10803	1858	0.17	EP300 (CH12 vs GM12878)	AdaLiftOver
46	33645	24466	2364	0.10	EP300 (CH12 vs GM12878)	bnMapper
47	33645	25215	2411	0.10	EP300 (CH12 vs GM12878)	UCSC liftOver
48	41335	14609	3117	0.21	EP300 (MEL vs K562)	AdaLiftOver
49	41335	27145	3028	0.11	EP300 (MEL vs K562)	bnMapper

Continued on next page

Supplementary Table 4 – continued from previous page

	# peaks	# map	# overlap	precision	sample	method
50	41335	28088	3120	0.11	EP300 (MEL vs K562)	UCSC liftOver
51	29200	11825	3068	0.26	ETS1 (CH12 vs GM12878)	AdaLiftOver
52	29200	21951	2540	0.12	ETS1 (CH12 vs GM12878)	bnMapper
53	29200	22658	2667	0.12	ETS1 (CH12 vs GM12878)	UCSC liftOver
54	38734	16858	4641	0.28	ETS1 (MEL vs K562)	AdaLiftOver
55	38734	26613	3440	0.13	ETS1 (MEL vs K562)	bnMapper
56	38734	27550	3593	0.13	ETS1 (MEL vs K562)	UCSC liftOver
57	45252	14359	1146	0.08	GATA1 (MEL vs K562)	AdaLiftOver
58	45252	28062	1413	0.05	GATA1 (MEL vs K562)	bnMapper
59	45252	28968	1428	0.05	GATA1 (MEL vs K562)	UCSC liftOver
60	41363	18562	3853	0.21	IRF4 (CH12 vs GM12878)	AdaLiftOver
61	41363	31104	3918	0.13	IRF4 (CH12 vs GM12878)	bnMapper
62	41363	32142	4040	0.13	IRF4 (CH12 vs GM12878)	UCSC liftOver
63	15762	5550	618	0.11	JUND (CH12 vs GM12878)	AdaLiftOver
64	15762	11411	750	0.07	JUND (CH12 vs GM12878)	bnMapper
65	15762	11786	765	0.06	JUND (CH12 vs GM12878)	UCSC liftOver
66	6674	2818	1073	0.38	JUND (MEL vs K562)	AdaLiftOver
67	6674	4355	988	0.23	JUND (MEL vs K562)	bnMapper
68	6674	4510	1017	0.23	JUND (MEL vs K562)	UCSC liftOver
69	10569	7685	74	0.01	KAT2A (CH12 vs GM12878)	AdaLiftOver
70	10569	8726	60	0.01	KAT2A (CH12 vs GM12878)	bnMapper
71	10569	9152	63	0.01	KAT2A (CH12 vs GM12878)	UCSC liftOver
72	2351	943	281	0.30	MAFK (MEL vs K562)	AdaLiftOver
73	2351	1479	328	0.22	MAFK (MEL vs K562)	bnMapper
74	2351	1525	333	0.22	MAFK (MEL vs K562)	UCSC liftOver
75	27057	15319	5860	0.38	MAX (CH12 vs GM12878)	AdaLiftOver
76	27057	20988	4783	0.23	MAX (CH12 vs GM12878)	bnMapper
77	27057	21865	5023	0.23	MAX (CH12 vs GM12878)	UCSC liftOver
78	24511	14527	8186	0.56	MAX (MEL vs K562)	AdaLiftOver
79	24511	18210	6446	0.35	MAX (MEL vs K562)	bnMapper
80	24511	19038	6787	0.36	MAX (MEL vs K562)	UCSC liftOver
81	17739	11632	6426	0.55	MAZ (CH12 vs GM12878)	AdaLiftOver
82	17739	14709	5622	0.38	MAZ (CH12 vs GM12878)	bnMapper
83	17739	15291	5899	0.39	MAZ (CH12 vs GM12878)	UCSC liftOver
84	16851	11707	7695	0.66	MAZ (MEL vs K562)	AdaLiftOver
85	16851	13589	6631	0.49	MAZ (MEL vs K562)	bnMapper
86	16851	14166	6949	0.49	MAZ (MEL vs K562)	UCSC liftOver
87	28178	13764	3342	0.24	MEF2A (CH12 vs GM12878)	AdaLiftOver
88	28178	21854	3511	0.16	MEF2A (CH12 vs GM12878)	bnMapper
89	28178	22638	3637	0.16	MEF2A (CH12 vs GM12878)	UCSC liftOver
90	4620	2065	294	0.14	MEF2A (MEL vs K562)	AdaLiftOver
91	4620	3361	301	0.09	MEF2A (MEL vs K562)	bnMapper
92	4620	3467	306	0.09	MEF2A (MEL vs K562)	UCSC liftOver
93	23755	16096	8317	0.52	MXI1 (CH12 vs GM12878)	AdaLiftOver
94	23755	19488	6960	0.36	MXI1 (CH12 vs GM12878)	bnMapper
95	23755	20445	7400	0.36	MXI1 (CH12 vs GM12878)	UCSC liftOver
96	29509	18473	4588	0.25	MXI1 (MEL vs K562)	AdaLiftOver
97	29509	22747	3529	0.16	MXI1 (MEL vs K562)	bnMapper

Continued on next page

Supplementary Table 4 – continued from previous page

	# peaks	# map	# overlap	precision	sample	method
98	29509	23869	3786	0.16	MXI1 (MEL vs K562)	UCSC liftOver
99	26233	14407	2517	0.17	MYC (CH12 vs GM12878)	AdaLiftOver
100	26233	19896	1860	0.09	MYC (CH12 vs GM12878)	bnMapper
101	26233	20648	1967	0.10	MYC (CH12 vs GM12878)	UCSC liftOver
102	23675	13283	7219	0.54	MYC (MEL vs K562)	AdaLiftOver
103	23675	17136	5759	0.34	MYC (MEL vs K562)	bnMapper
104	23675	17897	6055	0.34	MYC (MEL vs K562)	UCSC liftOver
105	16163	11913	3667	0.31	NRF1 (CH12 vs GM12878)	AdaLiftOver
106	16163	13206	3173	0.24	NRF1 (CH12 vs GM12878)	bnMapper
107	16163	13823	3354	0.24	NRF1 (CH12 vs GM12878)	UCSC liftOver
108	10599	8591	2700	0.31	NRF1 (MEL vs K562)	AdaLiftOver
109	10599	8861	2309	0.26	NRF1 (MEL vs K562)	bnMapper
110	10599	9254	2435	0.26	NRF1 (MEL vs K562)	UCSC liftOver
111	194	58	23	0.40	PAX5 (CH12 vs GM12878)	AdaLiftOver
112	194	119	19	0.16	PAX5 (CH12 vs GM12878)	bnMapper
113	194	129	22	0.17	PAX5 (CH12 vs GM12878)	UCSC liftOver
114	23696	16595	10486	0.63	POLR2A (CH12 vs GM12878)	AdaLiftOver
115	23696	18700	9080	0.49	POLR2A (CH12 vs GM12878)	bnMapper
116	23696	19889	9771	0.49	POLR2A (CH12 vs GM12878)	UCSC liftOver
117	21898	16012	3375	0.21	POLR2A (MEL vs K562)	AdaLiftOver
118	21898	16949	2761	0.16	POLR2A (MEL vs K562)	bnMapper
119	21898	18123	2943	0.16	POLR2A (MEL vs K562)	UCSC liftOver
120	41545	16323	10413	0.64	RAD21 (CH12 vs GM12878)	AdaLiftOver
121	41545	26278	12384	0.47	RAD21 (CH12 vs GM12878)	bnMapper
122	41545	27311	12736	0.47	RAD21 (CH12 vs GM12878)	UCSC liftOver
123	30908	14131	7430	0.53	RAD21 (MEL vs K562)	AdaLiftOver
124	30908	19912	8258	0.41	RAD21 (MEL vs K562)	bnMapper
125	30908	20695	8470	0.41	RAD21 (MEL vs K562)	UCSC liftOver
126	10893	5326	918	0.17	RCOR1 (CH12 vs GM12878)	AdaLiftOver
127	10893	8549	893	0.10	RCOR1 (CH12 vs GM12878)	bnMapper
128	10893	8877	929	0.10	RCOR1 (CH12 vs GM12878)	UCSC liftOver
129	25999	9929	678	0.07	RCOR1 (MEL vs K562)	AdaLiftOver
130	25999	17522	663	0.04	RCOR1 (MEL vs K562)	bnMapper
131	25999	18154	685	0.04	RCOR1 (MEL vs K562)	UCSC liftOver
132	17422	13091	397	0.03	RDBP (MEL vs K562)	AdaLiftOver
133	17422	14093	311	0.02	RDBP (MEL vs K562)	bnMapper
134	17422	14906	345	0.02	RDBP (MEL vs K562)	UCSC liftOver
135	27103	17821	7015	0.39	SIN3A (CH12 vs GM12878)	AdaLiftOver
136	27103	22261	5693	0.26	SIN3A (CH12 vs GM12878)	bnMapper
137	27103	23341	6077	0.26	SIN3A (CH12 vs GM12878)	UCSC liftOver
138	37908	22270	8181	0.37	SIN3A (MEL vs K562)	AdaLiftOver
139	37908	28786	6175	0.21	SIN3A (MEL vs K562)	bnMapper
140	37908	30118	6583	0.22	SIN3A (MEL vs K562)	UCSC liftOver
141	42669	17756	10953	0.62	SMC3 (CH12 vs GM12878)	AdaLiftOver
142	42669	28251	12299	0.44	SMC3 (CH12 vs GM12878)	bnMapper
143	42669	29363	12679	0.43	SMC3 (CH12 vs GM12878)	UCSC liftOver
144	39493	16863	9277	0.55	SMC3 (MEL vs K562)	AdaLiftOver
145	39493	23913	9838	0.41	SMC3 (MEL vs K562)	bnMapper

Continued on next page

Supplementary Table 4 – continued from previous page

	# peaks	# map	# overlap	precision	sample	method
146	39493	24866	10116	0.41	SMC3 (MEL vs K562)	UCSC liftOver
147	18204	6135	1045	0.17	TAL1 (MEL vs K562)	AdaLiftOver
148	18204	11767	1412	0.12	TAL1 (MEL vs K562)	bnMapper
149	18204	12163	1431	0.12	TAL1 (MEL vs K562)	UCSC liftOver
150	20458	12056	5799	0.48	TBP (CH12 vs GM12878)	AdaLiftOver
151	20458	15237	4950	0.32	TBP (CH12 vs GM12878)	bnMapper
152	20458	15969	5222	0.33	TBP (CH12 vs GM12878)	UCSC liftOver
153	26567	15495	7807	0.50	TBP (MEL vs K562)	AdaLiftOver
154	26567	18412	6140	0.33	TBP (MEL vs K562)	bnMapper
155	26567	19244	6447	0.34	TBP (MEL vs K562)	UCSC liftOver
156	32474	18158	6907	0.38	TCF12 (CH12 vs GM12878)	AdaLiftOver
157	32474	25471	6162	0.24	TCF12 (CH12 vs GM12878)	bnMapper
158	32474	26585	6476	0.24	TCF12 (CH12 vs GM12878)	UCSC liftOver
159	5111	4406	1811	0.41	UBTF (MEL vs K562)	AdaLiftOver
160	5111	4436	1470	0.33	UBTF (MEL vs K562)	bnMapper
161	5111	4691	1562	0.33	UBTF (MEL vs K562)	UCSC liftOver
162	8086	4116	1691	0.41	USF1 (CH12 vs GM12878)	AdaLiftOver
163	8086	6028	1612	0.27	USF1 (CH12 vs GM12878)	bnMapper
164	8086	6221	1660	0.27	USF1 (CH12 vs GM12878)	UCSC liftOver
165	19341	9449	3071	0.33	USF1 (MEL vs K562)	AdaLiftOver
166	19341	13293	2733	0.21	USF1 (MEL vs K562)	bnMapper
167	19341	13795	2838	0.21	USF1 (MEL vs K562)	UCSC liftOver
168	5662	2822	1036	0.37	USF2 (CH12 vs GM12878)	AdaLiftOver
169	5662	4266	981	0.23	USF2 (CH12 vs GM12878)	bnMapper
170	5662	4389	1010	0.23	USF2 (CH12 vs GM12878)	UCSC liftOver
171	1310	863	514	0.60	USF2 (MEL vs K562)	AdaLiftOver
172	1310	1030	504	0.49	USF2 (MEL vs K562)	bnMapper
173	1310	1062	520	0.49	USF2 (MEL vs K562)	UCSC liftOver

Supplementary Table 4: Benchmarking AdaLiftOver with the TF ChIP-seq datasets. Precision is computed as # of overlap divided by # of mapped.

	# peaks	# map	# overlap	precision	sample	method
1	20701	7035	4469	0.64	CTCF (spleen vs spleen)	AdaLiftOver
2	20701	14743	7746	0.53	CTCF (spleen vs spleen)	bnMapper
3	20701	14774	7914	0.54	CTCF (spleen vs spleen)	UCSC liftOver
4	74311	5460	1332	0.24	CTCF (stomach vs stomach)	AdaLiftOver
5	74311	49803	14961	0.30	CTCF (stomach vs stomach)	bnMapper
6	74311	49953	15657	0.31	CTCF (stomach vs stomach)	UCSC liftOver
7	24393	8093	5446	0.67	CTCF (testis vs testis)	AdaLiftOver
8	24393	16430	8718	0.53	CTCF (testis vs testis)	bnMapper
9	24393	16476	8984	0.55	CTCF (testis vs testis)	UCSC liftOver
10	6079	5916	512	0.09	EP300 (stomach vs stomach)	AdaLiftOver
11	6079	5509	507	0.09	EP300 (stomach vs stomach)	bnMapper
12	6079	5533	564	0.10	EP300 (stomach vs stomach)	UCSC liftOver
13	48349	24625	16168	0.66	H3K27ac (liver vs liver)	AdaLiftOver
14	48349	37447	16846	0.45	H3K27ac (liver vs liver)	bnMapper
15	48349	38030	18242	0.48	H3K27ac (liver vs liver)	UCSC liftOver
16	58076	30460	20461	0.67	H3K27ac (lung vs lung)	AdaLiftOver
17	58076	50822	22048	0.43	H3K27ac (lung vs lung)	bnMapper
18	58076	51287	23921	0.47	H3K27ac (lung vs lung)	UCSC liftOver
19	87631	35292	21013	0.60	H3K27ac (placenta vs placenta)	AdaLiftOver
20	87631	64448	23565	0.37	H3K27ac (placenta vs placenta)	bnMapper
21	87631	64812	26321	0.41	H3K27ac (placenta vs placenta)	UCSC liftOver
22	67664	40568	29119	0.72	H3K27ac (small intestine vs small intestine)	AdaLiftOver
23	67664	53611	29269	0.55	H3K27ac (small intestine vs small intestine)	bnMapper
24	67664	54001	31737	0.59	H3K27ac (small intestine vs small intestine)	UCSC liftOver
25	46259	25388	17815	0.70	H3K27ac (spleen vs spleen)	AdaLiftOver
26	46259	36702	18304	0.50	H3K27ac (spleen vs spleen)	bnMapper
27	46259	36969	19747	0.53	H3K27ac (spleen vs spleen)	UCSC liftOver
28	53097	36290	27284	0.75	H3K27ac (stomach vs stomach)	AdaLiftOver
29	53097	46325	27723	0.60	H3K27ac (stomach vs stomach)	bnMapper
30	53097	46681	28833	0.62	H3K27ac (stomach vs stomach)	UCSC liftOver
31	44156	24837	18327	0.74	H3K27ac (testis vs testis)	AdaLiftOver
32	44156	35097	18406	0.52	H3K27ac (testis vs testis)	bnMapper
33	44156	35316	19912	0.56	H3K27ac (testis vs testis)	UCSC liftOver
34	26724	14715	10566	0.72	H3K27ac (thymus vs thymus)	AdaLiftOver
35	26724	20787	10877	0.52	H3K27ac (thymus vs thymus)	bnMapper
36	26724	20963	11819	0.56	H3K27ac (thymus vs thymus)	UCSC liftOver
37	53033	5709	2565	0.45	H3K27me3 (heart vs heart)	AdaLiftOver
38	53033	42154	5290	0.13	H3K27me3 (heart vs heart)	bnMapper
39	53033	42849	6312	0.15	H3K27me3 (heart vs heart)	UCSC liftOver
40	47485	6501	2741	0.42	H3K27me3 (kidney vs kidney)	AdaLiftOver
41	47485	36347	5028	0.14	H3K27me3 (kidney vs kidney)	bnMapper
42	47485	36814	5932	0.16	H3K27me3 (kidney vs kidney)	UCSC liftOver
43	59326	2576	1023	0.40	H3K27me3 (liver vs liver)	AdaLiftOver
44	59326	43814	3897	0.09	H3K27me3 (liver vs liver)	bnMapper
45	59326	44559	4652	0.10	H3K27me3 (liver vs liver)	UCSC liftOver
46	22174	1990	740	0.37	H3K27me3 (lung vs lung)	AdaLiftOver

Continued on next page

Supplementary Table 5 – continued from previous page

	# peaks	# map	# overlap	precision	sample	method
47	22174	18185	1829	0.10	H3K27me3 (lung vs lung)	bnMapper
48	22174	18427	2077	0.11	H3K27me3 (lung vs lung)	UCSC liftOver
49	13291	11002	8425	0.77	H3K27me3 (small intestine vs small intestine)	AdaLiftOver
50	13291	11580	8197	0.71	H3K27me3 (small intestine vs small intestine)	bnMapper
51	13291	11630	8633	0.74	H3K27me3 (small intestine vs small intestine)	UCSC liftOver
52	13822	13589	1681	0.12	H3K27me3 (spleen vs spleen)	AdaLiftOver
53	13822	12742	1637	0.13	H3K27me3 (spleen vs spleen)	bnMapper
54	13822	12761	2198	0.17	H3K27me3 (spleen vs spleen)	UCSC liftOver
55	15476	13417	10395	0.77	H3K27me3 (stomach vs stomach)	AdaLiftOver
56	15476	13510	9917	0.73	H3K27me3 (stomach vs stomach)	bnMapper
57	15476	13608	10319	0.76	H3K27me3 (stomach vs stomach)	UCSC liftOver
58	1438	1427	55	0.04	H3K27me3 (testis vs testis)	AdaLiftOver
59	1438	1401	53	0.04	H3K27me3 (testis vs testis)	bnMapper
60	1438	1402	74	0.05	H3K27me3 (testis vs testis)	UCSC liftOver
61	8547	7870	6186	0.79	H3K27me3 (thymus vs thymus)	AdaLiftOver
62	8547	7904	6044	0.76	H3K27me3 (thymus vs thymus)	bnMapper
63	8547	7915	6323	0.80	H3K27me3 (thymus vs thymus)	UCSC liftOver
64	108803	7046	3202	0.45	H3K36me3 (heart vs heart)	AdaLiftOver
65	108803	80093	8559	0.11	H3K36me3 (heart vs heart)	bnMapper
66	108803	82146	11611	0.14	H3K36me3 (heart vs heart)	UCSC liftOver
67	108924	16941	8549	0.50	H3K36me3 (kidney vs kidney)	AdaLiftOver
68	108924	82303	13040	0.16	H3K36me3 (kidney vs kidney)	bnMapper
69	108924	84459	16874	0.20	H3K36me3 (kidney vs kidney)	UCSC liftOver
70	104288	49186	28110	0.57	H3K36me3 (liver vs liver)	AdaLiftOver
71	104288	76899	27691	0.36	H3K36me3 (liver vs liver)	bnMapper
72	104288	79115	33484	0.42	H3K36me3 (liver vs liver)	UCSC liftOver
73	85101	63998	37990	0.59	H3K36me3 (lung vs lung)	AdaLiftOver
74	85101	64563	30659	0.47	H3K36me3 (lung vs lung)	bnMapper
75	85101	65871	38156	0.58	H3K36me3 (lung vs lung)	UCSC liftOver
76	181271	35321	13285	0.38	H3K36me3 (small intestine vs small intestine)	AdaLiftOver
77	181271	124086	22669	0.18	H3K36me3 (small intestine vs small intestine)	bnMapper
78	181271	125187	32394	0.26	H3K36me3 (small intestine vs small intestine)	UCSC liftOver
79	124477	326	42	0.13	H3K36me3 (spleen vs spleen)	AdaLiftOver
80	124477	87441	5618	0.06	H3K36me3 (spleen vs spleen)	bnMapper
81	124477	88007	8272	0.09	H3K36me3 (spleen vs spleen)	UCSC liftOver
82	70664	63168	56707	0.90	H3K36me3 (stomach vs stomach)	AdaLiftOver
83	70664	52563	44796	0.85	H3K36me3 (stomach vs stomach)	bnMapper
84	70664	53293	46832	0.88	H3K36me3 (stomach vs stomach)	UCSC liftOver
85	13859	951	11	0.01	H3K36me3 (testis vs testis)	AdaLiftOver
86	13859	9620	44	0.00	H3K36me3 (testis vs testis)	bnMapper
87	13859	9650	72	0.01	H3K36me3 (testis vs testis)	UCSC liftOver
88	56413	52961	42185	0.80	H3K36me3 (thymus vs thymus)	AdaLiftOver

Continued on next page

Supplementary Table 5 – continued from previous page

	# peaks	# map	# overlap	precision	sample	method
89	56413	45472	34525	0.76	H3K36me3 (thymus vs thymus)	bnMapper
90	56413	46428	37798	0.81	H3K36me3 (thymus vs thymus)	UCSC liftOver
91	147429	44801	18643	0.42	H3K4me1 (heart vs heart)	AdaLiftOver
92	147429	121473	31666	0.26	H3K4me1 (heart vs heart)	bnMapper
93	147429	122555	35928	0.29	H3K4me1 (heart vs heart)	UCSC liftOver
94	142555	1594	308	0.19	H3K4me1 (kidney vs kidney)	AdaLiftOver
95	142555	118411	7618	0.06	H3K4me1 (kidney vs kidney)	bnMapper
96	142555	119290	9452	0.08	H3K4me1 (kidney vs kidney)	UCSC liftOver
97	102068	44916	25627	0.57	H3K4me1 (liver vs liver)	AdaLiftOver
98	102068	78086	27912	0.36	H3K4me1 (liver vs liver)	bnMapper
99	102068	79390	31514	0.40	H3K4me1 (liver vs liver)	UCSC liftOver
100	111510	41104	18160	0.44	H3K4me1 (lung vs lung)	AdaLiftOver
101	111510	94619	24597	0.26	H3K4me1 (lung vs lung)	bnMapper
102	111510	95324	30400	0.32	H3K4me1 (lung vs lung)	UCSC liftOver
103	109817	45281	24102	0.53	H3K4me1 (placenta vs placenta)	AdaLiftOver
104	109817	79597	29077	0.37	H3K4me1 (placenta vs placenta)	bnMapper
105	109817	79961	33156	0.41	H3K4me1 (placenta vs placenta)	UCSC liftOver
106	55897	812	168	0.21	H3K4me1 (small intestine vs small intestine)	AdaLiftOver
107	55897	41803	6118	0.15	H3K4me1 (small intestine vs small intestine)	bnMapper
108	55897	42030	8234	0.20	H3K4me1 (small intestine vs small intestine)	UCSC liftOver
109	28445	12241	6499	0.53	H3K4me1 (spleen vs spleen)	AdaLiftOver
110	28445	21377	7885	0.37	H3K4me1 (spleen vs spleen)	bnMapper
111	28445	21486	9295	0.43	H3K4me1 (spleen vs spleen)	UCSC liftOver
112	62869	50040	39101	0.78	H3K4me1 (stomach vs stomach)	AdaLiftOver
113	62869	53862	36747	0.68	H3K4me1 (stomach vs stomach)	bnMapper
114	62869	54133	38622	0.71	H3K4me1 (stomach vs stomach)	UCSC liftOver
115	51879	17053	7656	0.45	H3K4me1 (testis vs testis)	AdaLiftOver
116	51879	40286	12191	0.30	H3K4me1 (testis vs testis)	bnMapper
117	51879	40463	14785	0.37	H3K4me1 (testis vs testis)	UCSC liftOver
118	92393	25993	12246	0.47	H3K4me1 (thymus vs thymus)	AdaLiftOver
119	92393	66601	18757	0.28	H3K4me1 (thymus vs thymus)	bnMapper
120	92393	67043	22291	0.33	H3K4me1 (thymus vs thymus)	UCSC liftOver
121	33069	15822	10439	0.66	H3K4me3 (heart vs heart)	AdaLiftOver
122	33069	29663	11570	0.39	H3K4me3 (heart vs heart)	bnMapper
123	33069	30065	12397	0.41	H3K4me3 (heart vs heart)	UCSC liftOver
124	25091	17270	13310	0.77	H3K4me3 (kidney vs kidney)	AdaLiftOver
125	25091	22801	13812	0.61	H3K4me3 (kidney vs kidney)	bnMapper
126	25091	23160	14471	0.62	H3K4me3 (kidney vs kidney)	UCSC liftOver
127	24533	14960	11269	0.75	H3K4me3 (liver vs liver)	AdaLiftOver
128	24533	21634	11583	0.54	H3K4me3 (liver vs liver)	bnMapper
129	24533	22038	12135	0.55	H3K4me3 (liver vs liver)	UCSC liftOver
130	22754	15971	12701	0.80	H3K4me3 (lung vs lung)	AdaLiftOver
131	22754	20798	12926	0.62	H3K4me3 (lung vs lung)	bnMapper
132	22754	21116	13393	0.63	H3K4me3 (lung vs lung)	UCSC liftOver
133	16300	14398	12822	0.89	H3K4me3 (placenta vs placenta)	AdaLiftOver

Continued on next page

Supplementary Table 5 – continued from previous page

	# peaks	# map	# overlap	precision	sample	method
134	16300	15374	12999	0.85	H3K4me3 (placenta vs placenta)	bnMapper
135	16300	15458	13304	0.86	H3K4me3 (placenta vs placenta)	UCSC liftOver
136	33054	20188	14471	0.72	H3K4me3 (small intestine vs small intestine)	AdaLiftOver
137	33054	28969	14389	0.50	H3K4me3 (small intestine vs small intestine)	bnMapper
138	33054	29216	15631	0.54	H3K4me3 (small intestine vs small intestine)	UCSC liftOver
139	24957	19823	16824	0.85	H3K4me3 (spleen vs spleen)	AdaLiftOver
140	24957	22187	16221	0.73	H3K4me3 (spleen vs spleen)	bnMapper
141	24957	22357	17163	0.77	H3K4me3 (spleen vs spleen)	UCSC liftOver
142	23385	19506	17180	0.88	H3K4me3 (stomach vs stomach)	AdaLiftOver
143	23385	21439	16951	0.79	H3K4me3 (stomach vs stomach)	bnMapper
144	23385	21740	17420	0.80	H3K4me3 (stomach vs stomach)	UCSC liftOver
145	58275	26984	19438	0.72	H3K4me3 (testis vs testis)	AdaLiftOver
146	58275	42998	18917	0.44	H3K4me3 (testis vs testis)	bnMapper
147	58275	43547	20007	0.46	H3K4me3 (testis vs testis)	UCSC liftOver
148	24191	19759	17562	0.89	H3K4me3 (thymus vs thymus)	AdaLiftOver
149	24191	21595	17252	0.80	H3K4me3 (thymus vs thymus)	bnMapper
150	24191	21749	17679	0.81	H3K4me3 (thymus vs thymus)	UCSC liftOver
151	42875	30009	21216	0.71	H3K9ac (heart vs heart)	AdaLiftOver
152	42875	37645	21610	0.57	H3K9ac (heart vs heart)	bnMapper
153	42875	37931	23045	0.61	H3K9ac (heart vs heart)	UCSC liftOver
154	23426	16830	12932	0.77	H3K9ac (kidney vs kidney)	AdaLiftOver
155	23426	21654	13055	0.60	H3K9ac (kidney vs kidney)	bnMapper
156	23426	21857	13997	0.64	H3K9ac (kidney vs kidney)	UCSC liftOver
157	32198	16984	11774	0.69	H3K9ac (liver vs liver)	AdaLiftOver
158	32198	26455	11691	0.44	H3K9ac (liver vs liver)	bnMapper
159	32198	26844	12899	0.48	H3K9ac (liver vs liver)	UCSC liftOver
160	23821	18860	14543	0.77	H3K9ac (lung vs lung)	AdaLiftOver
161	23821	21905	14536	0.66	H3K9ac (lung vs lung)	bnMapper
162	23821	22098	15706	0.71	H3K9ac (lung vs lung)	UCSC liftOver
163	17382	118	46	0.39	H3K9me3 (heart vs heart)	AdaLiftOver
164	17382	2959	67	0.02	H3K9me3 (heart vs heart)	bnMapper
165	17382	3052	106	0.03	H3K9me3 (heart vs heart)	UCSC liftOver
166	15415	165	71	0.43	H3K9me3 (kidney vs kidney)	AdaLiftOver
167	15415	2852	106	0.04	H3K9me3 (kidney vs kidney)	bnMapper
168	15415	2910	157	0.05	H3K9me3 (kidney vs kidney)	UCSC liftOver
169	24183	260	131	0.50	H3K9me3 (liver vs liver)	AdaLiftOver
170	24183	7005	352	0.05	H3K9me3 (liver vs liver)	bnMapper
171	24183	7162	429	0.06	H3K9me3 (liver vs liver)	UCSC liftOver
172	3706	236	120	0.51	H3K9me3 (lung vs lung)	AdaLiftOver
173	3706	773	151	0.20	H3K9me3 (lung vs lung)	bnMapper
174	3706	785	207	0.26	H3K9me3 (lung vs lung)	UCSC liftOver
175	1191	171	114	0.67	H3K9me3 (stomach vs stomach)	AdaLiftOver
176	1191	325	105	0.32	H3K9me3 (stomach vs stomach)	bnMapper
177	1191	326	108	0.33	H3K9me3 (stomach vs stomach)	UCSC liftOver
178	160388	15613	8889	0.57	(heart vs heart)	AdaLiftOver

Continued on next page

Supplementary Table 5 – continued from previous page

	# peaks	# map	# overlap	precision	sample	method
179	160388	97874	16958	0.17	(heart vs heart)	bnMapper
180	160388	98171	20020	0.20	(heart vs heart)	UCSC liftOver
181	167449	29698	16066	0.54	(kidney vs kidney)	AdaLiftOver
182	167449	134566	29321	0.22	(kidney vs kidney)	bnMapper
183	167449	134807	35026	0.26	(kidney vs kidney)	UCSC liftOver
184	181924	19216	10031	0.52	(lung vs lung)	AdaLiftOver
185	181924	139455	27629	0.20	(lung vs lung)	bnMapper
186	181924	139730	33879	0.24	(lung vs lung)	UCSC liftOver
187	36640	16837	12225	0.73	(spleen vs spleen)	AdaLiftOver
188	36640	28232	13728	0.49	(spleen vs spleen)	bnMapper
189	36640	28291	15258	0.54	(spleen vs spleen)	UCSC liftOver
190	102729	36714	23787	0.65	(stomach vs stomach)	AdaLiftOver
191	102729	78361	32318	0.41	(stomach vs stomach)	bnMapper
192	102729	78519	36524	0.47	(stomach vs stomach)	UCSC liftOver
193	112029	13696	7662	0.56	(thymus vs thymus)	AdaLiftOver
194	112029	80121	17230	0.22	(thymus vs thymus)	bnMapper
195	112029	80343	19518	0.24	(thymus vs thymus)	UCSC liftOver
196	9722	1024	456	0.45	POLR2A (spleen vs spleen)	AdaLiftOver
197	9722	8938	2810	0.31	POLR2A (spleen vs spleen)	bnMapper
198	9722	8953	3389	0.38	POLR2A (spleen vs spleen)	UCSC liftOver
199	17357	2652	1219	0.46	POLR2A (testis vs testis)	AdaLiftOver
200	17357	14970	3496	0.23	POLR2A (testis vs testis)	bnMapper
201	17357	15058	3910	0.26	POLR2A (testis vs testis)	UCSC liftOver

Supplementary Table 5: The benchmarking results for 3 orthologous mapping methods with human-mouse dataset. *Number 10, 52, 58, 85 is calculated with threshold 0.01 rather than the default paramter 0.4 for AdaLiftOver due to the extremely low number of peaks in either target or query dataset.

	# peaks	# map	# overlap	precision	sample	method
1	146494	4870	3059	0.63	ATAC (brain vs brain)	AdaLiftOver
2	146494	18521	1689	0.09	ATAC (brain vs brain)	bnMapper
3	146494	21818	3403	0.16	ATAC (brain vs brain)	UCSC liftOver
4	68952	5080	3949	0.78	ATAC (colon vs colon)	AdaLiftOver
5	68952	8883	2008	0.23	ATAC (colon vs colon)	bnMapper
6	68952	10667	2958	0.28	ATAC (colon vs colon)	UCSC liftOver
7	142005	5274	3807	0.72	ATAC (heart vs heart)	AdaLiftOver
8	142005	18304	2182	0.12	ATAC (heart vs heart)	bnMapper
9	142005	21967	3523	0.16	ATAC (heart vs heart)	UCSC liftOver
10	118775	5689	3687	0.65	ATAC (intestine vs intestine)	AdaLiftOver
11	118775	15976	1839	0.12	ATAC (intestine vs intestine)	bnMapper
12	118775	18913	4032	0.21	ATAC (intestine vs intestine)	UCSC liftOver
13	151319	6733	4970	0.74	ATAC (kidney vs kidney)	AdaLiftOver
14	151319	20382	2893	0.14	ATAC (kidney vs kidney)	bnMapper
15	151319	24423	4445	0.18	ATAC (kidney vs kidney)	UCSC liftOver
16	99099	5956	4365	0.73	ATAC (liver vs liver)	AdaLiftOver
17	99099	11962	2092	0.17	ATAC (liver vs liver)	bnMapper
18	99099	14456	3300	0.23	ATAC (liver vs liver)	UCSC liftOver
19	109504	920	557	0.61	ATAC (muscle vs muscle)	AdaLiftOver
20	109504	13607	689	0.05	ATAC (muscle vs muscle)	bnMapper
21	109504	16175	1797	0.11	ATAC (muscle vs muscle)	UCSC liftOver
22	185631	375	195	0.52	ATAC (skin vs skin)	AdaLiftOver
23	185631	23265	617	0.03	ATAC (skin vs skin)	bnMapper
24	185631	28086	1803	0.06	ATAC (skin vs skin)	UCSC liftOver
25	108665	5318	3860	0.73	ATAC (spleen vs spleen)	AdaLiftOver
26	108665	14468	1891	0.13	ATAC (spleen vs spleen)	bnMapper
27	108665	17496	2934	0.17	ATAC (spleen vs spleen)	UCSC liftOver
28	152416	5767	3863	0.67	ATAC (testis vs testis)	AdaLiftOver
29	152417	21076	2299	0.11	ATAC (testis vs testis)	bnMapper
30	152417	25450	3916	0.15	ATAC (testis vs testis)	UCSC liftOver
31	33282	2776	2067	0.74	H3K27ac (brain vs brain)	AdaLiftOver
32	33282	4535	1169	0.26	H3K27ac (brain vs brain)	bnMapper
33	33282	5518	1632	0.30	H3K27ac (brain vs brain)	UCSC liftOver
34	64618	6707	5113	0.76	H3K27ac (colon vs colon)	AdaLiftOver
35	64618	8651	2977	0.34	H3K27ac (colon vs colon)	bnMapper
36	64618	10809	4140	0.38	H3K27ac (colon vs colon)	UCSC liftOver
37	57632	6368	4987	0.78	H3K27ac (heart vs heart)	AdaLiftOver
38	57632	6141	2378	0.39	H3K27ac (heart vs heart)	bnMapper
39	57632	7491	3180	0.42	H3K27ac (heart vs heart)	UCSC liftOver
40	50313	5399	4311	0.80	H3K27ac (intestine vs intestine)	AdaLiftOver
41	50313	6766	2276	0.34	H3K27ac (intestine vs intestine)	bnMapper
42	50313	8579	3252	0.38	H3K27ac (intestine vs intestine)	UCSC liftOver
43	121655	5810	4166	0.72	H3K27ac (kidney vs kidney)	AdaLiftOver
44	121655	15394	2419	0.16	H3K27ac (kidney vs kidney)	bnMapper
45	121655	18493	3610	0.20	H3K27ac (kidney vs kidney)	UCSC liftOver
46	79944	6459	4806	0.74	H3K27ac (liver vs liver)	AdaLiftOver
47	79944	10303	2549	0.25	H3K27ac (liver vs liver)	bnMapper
48	79944	12472	3669	0.29	H3K27ac (liver vs liver)	UCSC liftOver
49	107940	8263	6167	0.75	H3K27ac (muscle vs muscle)	AdaLiftOver

Continued on next page

Supplementary Table 6 – continued from previous page

	# peaks	# map	# overlap	precision	sample	method
50	107940	10594	2998	0.28	H3K27ac (muscle vs muscle)	bnMapper
51	107940	12648	4103	0.32	H3K27ac (muscle vs muscle)	UCSC liftOver
52	16937	3138	2785	0.89	H3K27ac (skin vs skin)	AdaLiftOver
53	16937	2774	1563	0.56	H3K27ac (skin vs skin)	bnMapper
54	16937	3421	2042	0.60	H3K27ac (skin vs skin)	UCSC liftOver
55	39160	4123	3283	0.80	H3K27ac (spleen vs spleen)	AdaLiftOver
56	39160	5411	1725	0.32	H3K27ac (spleen vs spleen)	bnMapper
57	39160	6636	2391	0.36	H3K27ac (spleen vs spleen)	UCSC liftOver
58	49799	4872	3839	0.79	H3K27ac (testis vs testis)	AdaLiftOver
59	49799	7249	1886	0.26	H3K27ac (testis vs testis)	bnMapper
60	49799	9039	2783	0.31	H3K27ac (testis vs testis)	UCSC liftOver
61	23015	5966	5202	0.87	H3K4me3 (brain vs brain)	AdaLiftOver
62	23015	4487	2914	0.65	H3K4me3 (brain vs brain)	bnMapper
63	23015	5637	3868	0.69	H3K4me3 (brain vs brain)	UCSC liftOver
64	40842	6204	5388	0.87	H3K4me3 (colon vs colon)	AdaLiftOver
65	40842	5854	2747	0.47	H3K4me3 (colon vs colon)	bnMapper
66	40842	7325	3732	0.51	H3K4me3 (colon vs colon)	UCSC liftOver
67	21759	4328	3197	0.74	H3K4me3 (heart vs heart)	AdaLiftOver
68	21759	4390	1274	0.29	H3K4me3 (heart vs heart)	bnMapper
69	21759	5653	2422	0.43	H3K4me3 (heart vs heart)	UCSC liftOver
70	30059	4755	3968	0.83	H3K4me3 (intestine vs intestine)	AdaLiftOver
71	30059	5002	2098	0.42	H3K4me3 (intestine vs intestine)	bnMapper
72	30059	6382	2988	0.47	H3K4me3 (intestine vs intestine)	UCSC liftOver
73	32734	5874	4801	0.82	H3K4me3 (kidney vs kidney)	AdaLiftOver
74	32734	5762	2305	0.40	H3K4me3 (kidney vs kidney)	bnMapper
75	32734	7298	3452	0.47	H3K4me3 (kidney vs kidney)	UCSC liftOver
76	26386	5009	4053	0.81	H3K4me3 (liver vs liver)	AdaLiftOver
77	26386	4806	2068	0.43	H3K4me3 (liver vs liver)	bnMapper
78	26386	6173	3087	0.50	H3K4me3 (liver vs liver)	UCSC liftOver
79	26538	6071	5296	0.87	H3K4me3 (muscle vs muscle)	AdaLiftOver
80	26538	4962	2768	0.56	H3K4me3 (muscle vs muscle)	bnMapper
81	26538	6313	3812	0.60	H3K4me3 (muscle vs muscle)	UCSC liftOver
82	22004	5319	4804	0.90	H3K4me3 (skin vs skin)	AdaLiftOver
83	22004	4368	2640	0.60	H3K4me3 (skin vs skin)	bnMapper
84	22004	5638	3629	0.64	H3K4me3 (skin vs skin)	UCSC liftOver
85	26152	5116	4369	0.85	H3K4me3 (spleen vs spleen)	AdaLiftOver
86	26152	5014	2316	0.46	H3K4me3 (spleen vs spleen)	bnMapper
87	26152	6551	3359	0.51	H3K4me3 (spleen vs spleen)	UCSC liftOver
88	39555	6039	4914	0.81	H3K4me3 (testis vs testis)	AdaLiftOver
89	39555	6074	2695	0.44	H3K4me3 (testis vs testis)	bnMapper
90	39555	7776	3870	0.50	H3K4me3 (testis vs testis)	UCSC liftOver

Supplementary Table 6: The benchmarking results for 3 orthologous mapping methods with human-zebrafish dataset.

	# peaks	# map	# overlap	precision	sample	method
1	21907	4807	2729	0.57	DNaseSeq (muscle vs muscle)	AdaLiftOver
2	21907	7449	2408	0.32	DNaseSeq (muscle vs muscle)	bnMapper
3	21907	9179	3763	0.41	DNaseSeq (muscle vs muscle)	UCSC liftOver
4	19890	8399	6745	0.80	H3K27ac (liver vs liver)	AdaLiftOver
5	19890	8004	4279	0.53	H3K27ac (liver vs liver)	bnMapper
6	19890	10722	6607	0.62	H3K27ac (liver vs liver)	UCSC liftOver
7	13072	7005	5890	0.84	H3K27ac (muscle vs muscle)	AdaLiftOver
8	13072	5858	3726	0.64	H3K27ac (muscle vs muscle)	bnMapper
9	13072	8672	6197	0.71	H3K27ac (muscle vs muscle)	UCSC liftOver
10	21960	8792	6802	0.77	H3K27ac (spleen vs spleen)	AdaLiftOver
11	21960	8485	4316	0.51	H3K27ac (spleen vs spleen)	bnMapper
12	21960	10876	6331	0.58	H3K27ac (spleen vs spleen)	UCSC liftOver
13	17292	9167	6989	0.76	H3K4me3 (liver vs liver)	AdaLiftOver
14	17292	7641	3900	0.51	H3K4me3 (liver vs liver)	bnMapper
15	17292	11657	7113	0.61	H3K4me3 (liver vs liver)	UCSC liftOver
16	12706	8719	8074	0.93	H3K4me3 (muscle vs muscle)	AdaLiftOver
17	12706	6288	4901	0.78	H3K4me3 (muscle vs muscle)	bnMapper
18	12706	9693	8122	0.84	H3K4me3 (muscle vs muscle)	UCSC liftOver
19	16894	10660	8956	0.84	H3K4me3 (spleen vs spleen)	AdaLiftOver
20	16894	8365	5515	0.66	H3K4me3 (spleen vs spleen)	bnMapper
21	16894	11753	8541	0.73	H3K4me3 (spleen vs spleen)	UCSC liftOver

Supplementary Table 7: The benchmarking results for 3 orthologous mapping methods with human-chicken dataset.

	# peaks	# map	# overlap	precision	sample	method
1	21907	318	136	0.43	ATAC (liver vs liver)	AdaLiftOver
2	21907	13240	500	0.04	ATAC (liver vs liver)	bnMapper
3	21907	13453	676	0.05	ATAC (liver vs liver)	UCSC liftOver
4	19890	303	127	0.42	ATAC (spleen vs spleen)	AdaLiftOver
5	19890	13098	404	0.03	ATAC (spleen vs spleen)	bnMapper
6	19890	13533	509	0.04	ATAC (spleen vs spleen)	UCSC liftOver
7	13072	548	284	0.52	H3K27ac (liver vs liver)	AdaLiftOver
8	13072	8776	514	0.06	H3K27ac (liver vs liver)	bnMapper
9	13072	9151	633	0.07	H3K27ac (liver vs liver)	UCSC liftOver
10	21960	626	314	0.50	H3K27ac (muscle vs muscle)	AdaLiftOver
11	21960	13688	866	0.06	H3K27ac (muscle vs muscle)	bnMapper
12	21960	14035	1027	0.07	H3K27ac (muscle vs muscle)	UCSC liftOver
13	17292	361	208	0.58	H3K27ac (spleen vs spleen)	AdaLiftOver
14	17292	11348	364	0.03	H3K27ac (spleen vs spleen)	bnMapper
15	17292	11738	430	0.04	H3K27ac (spleen vs spleen)	UCSC liftOver
16	12706	245	148	0.60	H3K4me3 (liver vs liver)	AdaLiftOver
17	12706	8201	211	0.03	H3K4me3 (liver vs liver)	bnMapper
18	12706	8459	256	0.03	H3K4me3 (liver vs liver)	UCSC liftOver
19	16894	489	301	0.62	H3K4me3 (muscle vs muscle)	AdaLiftOver
20	16894	10525	424	0.04	H3K4me3 (muscle vs muscle)	bnMapper
21	16894	10775	520	0.05	H3K4me3 (muscle vs muscle)	UCSC liftOver
22	58199	15829	7201	0.45	H3K4me3 (spleen vs spleen)	AdaLiftOver
23	58199	52337	13824	0.26	H3K4me3 (spleen vs spleen)	bnMapper
24	58199	52936	14136	0.27	H3K4me3 (spleen vs spleen)	UCSC liftOver

Supplementary Table 8: The benchmarking results for 3 orthologous mapping methods with human-cow dataset.

	# peaks	# map	# overlap	precision	sample	method
1	78788	29537	15669	0.53	H3K27ac (brain vs brain)	AdaLiftOver
2	78788	74948	19325	0.26	H3K27ac (brain vs brain)	bnMapper
3	78788	75619	21210	0.28	H3K27ac (brain vs brain)	UCSC liftOver
4	68684	42579	32924	0.77	H3K27ac (heart vs heart)	AdaLiftOver
5	68684	65040	38666	0.59	H3K27ac (heart vs heart)	bnMapper
6	68684	65669	40744	0.62	H3K27ac (heart vs heart)	UCSC liftOver
7	87557	41885	23925	0.57	H3K27ac (liver vs liver)	AdaLiftOver
8	87557	81352	31023	0.38	H3K27ac (liver vs liver)	bnMapper
9	87557	82157	34313	0.42	H3K27ac (liver vs liver)	UCSC liftOver
10	76453	44898	29593	0.66	H3K27ac (muscle vs muscle)	AdaLiftOver
11	76453	72006	34450	0.48	H3K27ac (muscle vs muscle)	bnMapper
12	76453	72648	37312	0.51	H3K27ac (muscle vs muscle)	UCSC liftOver
13	27094	23403	18851	0.81	H3K4me3 (brain vs brain)	AdaLiftOver
14	27094	25820	19083	0.74	H3K4me3 (brain vs brain)	bnMapper
15	27094	26271	19745	0.75	H3K4me3 (brain vs brain)	UCSC liftOver
16	26469	16619	11340	0.68	H3K4me3 (heart vs heart)	AdaLiftOver
17	26469	25257	12370	0.49	H3K4me3 (heart vs heart)	bnMapper
18	26469	25672	13138	0.51	H3K4me3 (heart vs heart)	UCSC liftOver
19	28494	20256	13270	0.66	H3K4me3 (liver vs liver)	AdaLiftOver
20	28494	26780	13665	0.51	H3K4me3 (liver vs liver)	bnMapper
21	28494	27276	14478	0.53	H3K4me3 (liver vs liver)	UCSC liftOver
22	28104	22805	18994	0.83	H3K4me3 (muscle vs muscle)	AdaLiftOver
23	28104	26815	19238	0.72	H3K4me3 (muscle vs muscle)	bnMapper
24	28104	27277	20216	0.74	H3K4me3 (muscle vs muscle)	UCSC liftOver

Supplementary Table 9: The benchmarking results for 3 orthologous mapping methods with human-horse dataset.

	# peaks	# map	# overlap	precision	sample	method
1	69542	25848	16943	0.66	ATAC (liver vs liver)	AdaLiftOver
2	69542	61376	22914	0.37	ATAC (liver vs liver)	bnMapper
3	69542	63938	25271	0.40	ATAC (liver vs liver)	UCSC liftOver
4	18463	12950	9529	0.74	H3K27ac (brain vs brain)	AdaLiftOver
5	18463	16606	9149	0.55	H3K27ac (brain vs brain)	bnMapper
6	18463	18183	10489	0.58	H3K27ac (brain vs brain)	UCSC liftOver
7	29441	21922	18961	0.86	H3K27ac (heart vs heart)	AdaLiftOver
8	29441	26677	19343	0.73	H3K27ac (heart vs heart)	bnMapper
9	29441	28616	21274	0.74	H3K27ac (heart vs heart)	UCSC liftOver
10	29721	21831	17503	0.80	H3K27ac (intestine vs intestine)	AdaLiftOver
11	29721	26423	17290	0.65	H3K27ac (intestine vs intestine)	bnMapper
12	29721	28740	19546	0.68	H3K27ac (intestine vs intestine)	UCSC liftOver
13	27773	19743	15072	0.76	H3K27ac (kidney vs kidney)	AdaLiftOver
14	27773	24838	14959	0.60	H3K27ac (kidney vs kidney)	bnMapper
15	27773	26969	16902	0.63	H3K27ac (kidney vs kidney)	UCSC liftOver
16	46061	23392	16470	0.70	H3K27ac (liver vs liver)	AdaLiftOver
17	46061	40701	18269	0.45	H3K27ac (liver vs liver)	bnMapper
18	46061	43456	20656	0.48	H3K27ac (liver vs liver)	UCSC liftOver
19	32101	22625	17799	0.79	H3K27ac (muscle vs muscle)	AdaLiftOver
20	32101	28981	18412	0.64	H3K27ac (muscle vs muscle)	bnMapper
21	32101	31056	20517	0.66	H3K27ac (muscle vs muscle)	UCSC liftOver
22	30072	17216	11941	0.69	H3K27ac (spleen vs spleen)	AdaLiftOver
23	30072	26480	11861	0.45	H3K27ac (spleen vs spleen)	bnMapper
24	30072	28771	13666	0.47	H3K27ac (spleen vs spleen)	UCSC liftOver
25	12359	12151	11903	0.98	H3K4me3 (brain vs brain)	AdaLiftOver
26	12359	10694	10468	0.98	H3K4me3 (brain vs brain)	bnMapper
27	12359	12182	11931	0.98	H3K4me3 (brain vs brain)	UCSC liftOver
28	12815	11485	9128	0.79	H3K4me3 (heart vs heart)	AdaLiftOver
29	12815	11129	8413	0.76	H3K4me3 (heart vs heart)	bnMapper
30	12815	12608	9684	0.77	H3K4me3 (heart vs heart)	UCSC liftOver
31	12953	11995	10770	0.90	H3K4me3 (intestine vs intestine)	AdaLiftOver
32	12953	11200	9704	0.87	H3K4me3 (intestine vs intestine)	bnMapper
33	12953	12739	11160	0.88	H3K4me3 (intestine vs intestine)	UCSC liftOver
34	11596	11178	10580	0.95	H3K4me3 (kidney vs kidney)	AdaLiftOver
35	11596	9998	9375	0.94	H3K4me3 (kidney vs kidney)	bnMapper
36	11596	11444	10759	0.94	H3K4me3 (kidney vs kidney)	UCSC liftOver
37	13265	12276	10470	0.85	H3K4me3 (liver vs liver)	AdaLiftOver
38	13265	11450	9467	0.83	H3K4me3 (liver vs liver)	bnMapper
39	13265	13040	10907	0.84	H3K4me3 (liver vs liver)	UCSC liftOver
40	13289	12812	12325	0.96	H3K4me3 (muscle vs muscle)	AdaLiftOver
41	13289	11512	10957	0.95	H3K4me3 (muscle vs muscle)	bnMapper
42	13289	13083	12476	0.95	H3K4me3 (muscle vs muscle)	UCSC liftOver
43	12575	11587	10799	0.93	H3K4me3 (spleen vs spleen)	AdaLiftOver
44	12575	10750	9644	0.90	H3K4me3 (spleen vs spleen)	bnMapper
45	12575	12331	11126	0.90	H3K4me3 (spleen vs spleen)	UCSC liftOver

Supplementary Table 10: The benchmarking results for 3 orthologous mapping methods with human-pig dataset.

	# peaks	# map	# overlap	precision	sample	method
1	39958	23202	19681	0.85	H3K4me3 (heart vs heart)	AdaLiftOver
2	39958	32283	19592	0.61	H3K4me3 (heart vs heart)	bnMapper
3	39958	33284	22776	0.68	H3K4me3 (heart vs heart)	UCSC liftOver
4	16980	12952	11778	0.91	H3K4me3 (liver vs liver)	AdaLiftOver
5	16980	13667	11477	0.84	H3K4me3 (liver vs liver)	bnMapper
6	16980	14607	12481	0.85	H3K4me3 (liver vs liver)	UCSC liftOver
7	37185	20351	11604	0.57	H3K4me3 (pancreas vs pancreas)	AdaLiftOver
8	37185	29093	11614	0.40	H3K4me3 (pancreas vs pancreas)	bnMapper
9	37185	30399	14084	0.46	H3K4me3 (pancreas vs pancreas)	UCSC liftOver

Supplementary Table 11: The benchmarking results for 3 orthologous mapping methods with human-rat dataset.

Methods	Precision: mean and s.e.																Run time	Memory
	ENCODE TF ChIP-seq		Chicken		Cow		Horse		Mouse		Pig		Rat		Zebrafish			
AdaLiftOver	0.38	0.023	0.79	0.042	0.51	0.027	0.69	0.038	0.60	0.022	0.82	0.026	0.78	0.104	0.76	0.016	∼20 minutes	3.4G
bnMapper (Denas *et al.*, 2015)	0.26	0.018	0.56	0.055	0.07	0.028	0.52	0.057	0.38	0.029	0.71	0.051	0.62	0.127	0.3	0.031	∼10 minutes	336M
EpiAlignment (Lu *et al.*, 2019)	0.23	0.027	-	-	-	-	-	-	-	-	-	-	-	-	-	-	∼3 hours	-(Run on WebApp)
UCSC liftOver (Hinrichs *et al.*, 2006)	0.26	0.019	0.64	0.051	0.08	0.027	0.54	0.055	0.42	0.029	0.73	0.048	0.67	0.113	0.35	0.031	∼10 seconds	1.6G

Supplementary Table 12: Comparison among existing orthologues mapping methods with case studies in Section 3.1. For each case study, mean and standard error of precision across all the benchmarking instances are reported. Values for EpiAlignment are calculated on a subset of the samples as noted in Section 3.1.

	# map	# overlap	precision	method	sample
1	612	16	0.03	AdaLiftOver	Microglia
2	612	12	0.02	AdaLiftOver	Astrocytes*
3	612	13	0.02	AdaLiftOver	Cones (Blue)
4	612	23	0.04	AdaLiftOver	CD4 T-cells
5	612	22	0.04	AdaLiftOver	CD8 T-cells
6	612	57	0.09	AdaLiftOver	Excitatory Camk2a
7	612	12	0.02	AdaLiftOver	Inhibitory PV
8	612	16	0.03	AdaLiftOver	Inhibitory VIP
9	612	105	0.17	AdaLiftOver	Excitatory Layers II-III
10	612	69	0.11	AdaLiftOver	Excitatory Layer VI*
11	612	97	0.16	AdaLiftOver	Excitatory DG*
12	612	105	0.17	AdaLiftOver	Excitatory Layers II-V*
13	612	21	0.03	AdaLiftOver	Embryonic DA forebrain
14	612	113	0.18	AdaLiftOver	Inhibitory Gad2
15	612	14	0.02	AdaLiftOver	Cones (Green)
16	612	64	0.10	AdaLiftOver	Inhibitory*
17	612	73	0.12	AdaLiftOver	Inhibitory MSN*
18	612	20	0.03	AdaLiftOver	Embryonic DA midbrain
19	612	12	0.02	AdaLiftOver	Microglia*
20	612	17	0.03	AdaLiftOver	Neun negative
21	612	117	0.19	AdaLiftOver	Excitatory Layer VI
22	612	11	0.02	AdaLiftOver	Oligodendrocytes*
23	612	113	0.18	AdaLiftOver	Excitatory Layer V
24	612	9	0.01	AdaLiftOver	Rods
25	612	103	0.17	AdaLiftOver	Excitatory Layer IV
26	681	8	0.01	bnMapper	Microglia
27	681	8	0.01	bnMapper	Astrocytes*
28	681	7	0.01	bnMapper	Cones (Blue)
29	681	14	0.02	bnMapper	CD4 T-cells
30	681	15	0.02	bnMapper	CD8 T-cells
31	681	71	0.10	bnMapper	Excitatory Camk2a
32	681	10	0.01	bnMapper	Inhibitory PV
33	681	12	0.02	bnMapper	Inhibitory VIP
34	681	102	0.15	bnMapper	Excitatory Layers II-III
35	681	75	0.11	bnMapper	Excitatory Layer VI*
36	681	108	0.16	bnMapper	Excitatory DG*
37	681	108	0.16	bnMapper	Excitatory Layers II-V*
38	681	16	0.02	bnMapper	Embryonic DA forebrain
39	681	97	0.14	bnMapper	Inhibitory Gad2
40	681	7	0.01	bnMapper	Cones (Green)
41	681	57	0.08	bnMapper	Inhibitory*
42	681	66	0.10	bnMapper	Inhibitory MSN*
43	681	14	0.02	bnMapper	Embryonic DA midbrain
44	681	5	0.01	bnMapper	Microglia*
45	681	12	0.02	bnMapper	Neun negative
46	681	107	0.16	bnMapper	Excitatory Layer VI
47	681	7	0.01	bnMapper	Oligodendrocytes*
48	681	105	0.15	bnMapper	Excitatory Layer V
49	681	5	0.01	bnMapper	Rods

Continued on next page

Supplementary Table 13 – continued from previous page

	# map	# overlap	precision	method	sample
50	681	94	0.14	bnMapper	Excitatory Layer IV
51	1209	9	0.01	EpiAlignment	Microglia
52	1209	12	0.01	EpiAlignment	Astrocytes*
53	1209	5	0.00	EpiAlignment	Cones (Blue)
54	1209	7	0.01	EpiAlignment	CD4 T-cells
55	1209	10	0.01	EpiAlignment	CD8 T-cells
56	1209	54	0.04	EpiAlignment	Excitatory Camk2a
57	1209	4	0.00	EpiAlignment	Inhibitory PV
58	1209	6	0.00	EpiAlignment	Inhibitory VIP
59	1209	100	0.08	EpiAlignment	Excitatory Layers II-III
60	1209	58	0.05	EpiAlignment	Excitatory Layer VI*
61	1209	89	0.07	EpiAlignment	Excitatory DG*
62	1209	76	0.06	EpiAlignment	Excitatory Layers II-V*
63	1209	7	0.01	EpiAlignment	Embryonic DA forebrain
64	1209	92	0.08	EpiAlignment	Inhibitory Gad2
65	1209	5	0.00	EpiAlignment	Cones (Green)
66	1209	38	0.03	EpiAlignment	Inhibitory*
67	1209	42	0.03	EpiAlignment	Inhibitory MSN*
68	1209	11	0.01	EpiAlignment	Embryonic DA midbrain
69	1209	5	0.00	EpiAlignment	Microglia*
70	1209	9	0.01	EpiAlignment	Neun negative
71	1209	114	0.09	EpiAlignment	Excitatory Layer VI
72	1209	4	0.00	EpiAlignment	Oligodendrocytes*
73	1209	104	0.09	EpiAlignment	Excitatory Layer V
74	1209	3	0.00	EpiAlignment	Rods
75	1209	100	0.08	EpiAlignment	Excitatory Layer IV
76	715	9	0.01	UCSC liftOver	Microglia
77	715	10	0.01	UCSC liftOver	Astrocytes*
78	715	9	0.01	UCSC liftOver	Cones (Blue)
79	715	16	0.02	UCSC liftOver	CD4 T-cells
80	715	16	0.02	UCSC liftOver	CD8 T-cells
81	715	71	0.10	UCSC liftOver	Excitatory Camk2a
82	715	12	0.02	UCSC liftOver	Inhibitory PV
83	715	13	0.02	UCSC liftOver	Inhibitory VIP
84	715	109	0.15	UCSC liftOver	Excitatory Layers II-III
85	715	78	0.11	UCSC liftOver	Excitatory Layer VI*
86	715	114	0.16	UCSC liftOver	Excitatory DG*
87	715	114	0.16	UCSC liftOver	Excitatory Layers II-V*
88	715	17	0.02	UCSC liftOver	Embryonic DA forebrain
89	715	102	0.14	UCSC liftOver	Inhibitory Gad2
90	715	7	0.01	UCSC liftOver	Cones (Green)
91	715	59	0.08	UCSC liftOver	Inhibitory*
92	715	66	0.09	UCSC liftOver	Inhibitory MSN*
93	715	15	0.02	UCSC liftOver	Embryonic DA midbrain
94	715	6	0.01	UCSC liftOver	Microglia*
95	715	12	0.02	UCSC liftOver	Neun negative
96	715	112	0.16	UCSC liftOver	Excitatory Layer VI
97	715	7	0.01	UCSC liftOver	Oligodendrocytes*

Continued on next page

Supplementary Table 13 – continued from previous page

	# map	# overlap	precision	method	sample
98	715	109	0.15	UCSC liftOver	Excitatory Layer V
99	715	5	0.01	UCSC liftOver	Rods
100	715	99	0.14	UCSC liftOver	Excitatory Layer IV

Supplementary Table 13: Benchmarking AdaLiftOver on 1,648 SCZ GWAS SNPs.

	# mapped	# overlapped	precision	method	cell type	trait
1	226	14	0.06	AdaLiftOver	B	MCV
2	226	12	0.05	AdaLiftOver	CD4	MCV
3	226	10	0.04	AdaLiftOver	CD8	MCV
4	226	39	0.17	AdaLiftOver	CMP	MCV
5	226	36	0.16	AdaLiftOver	ERY	MCV
6	226	33	0.15	AdaLiftOver	GMP	MCV
7	226	16	0.07	AdaLiftOver	Mega	MCV
8	226	35	0.15	AdaLiftOver	MEP	MCV
9	226	11	0.05	AdaLiftOver	Mono	MCV
10	226	14	0.06	AdaLiftOver	NK	MCV
11	217	12	0.06	bnMapper	B	MCV
12	217	9	0.04	bnMapper	CD4	MCV
13	217	5	0.02	bnMapper	CD8	MCV
14	217	36	0.17	bnMapper	CMP	MCV
15	217	28	0.13	bnMapper	ERY	MCV
16	217	27	0.12	bnMapper	GMP	MCV
17	217	18	0.08	bnMapper	Mega	MCV
18	217	36	0.17	bnMapper	MEP	MCV
19	217	7	0.03	bnMapper	Mono	MCV
20	217	11	0.05	bnMapper	NK	MCV
21	527	18	0.03	EpiAlignment	B	MCV
22	527	12	0.02	EpiAlignment	CD4	MCV
23	527	10	0.02	EpiAlignment	CD8	MCV
24	527	31	0.06	EpiAlignment	CMP	MCV
25	527	31	0.06	EpiAlignment	ERY	MCV
26	527	29	0.06	EpiAlignment	GMP	MCV
27	527	15	0.03	EpiAlignment	Mega	MCV
28	527	31	0.06	EpiAlignment	MEP	MCV
29	527	12	0.02	EpiAlignment	Mono	MCV
30	527	16	0.03	EpiAlignment	NK	MCV
31	228	11	0.05	UCSC liftOver	B	MCV
32	228	10	0.04	UCSC liftOver	CD4	MCV
33	228	6	0.03	UCSC liftOver	CD8	MCV
34	228	39	0.17	UCSC liftOver	CMP	MCV
35	228	30	0.13	UCSC liftOver	ERY	MCV
36	228	29	0.13	UCSC liftOver	GMP	MCV
37	228	19	0.08	UCSC liftOver	Mega	MCV
38	228	38	0.17	UCSC liftOver	MEP	MCV
39	228	9	0.04	UCSC liftOver	Mono	MCV
40	228	11	0.05	UCSC liftOver	NK	MCV
41	271	19	0.07	AdaLiftOver	B	MPV
42	271	21	0.08	AdaLiftOver	CD4	MPV
43	271	22	0.08	AdaLiftOver	CD8	MPV
44	271	53	0.20	AdaLiftOver	CMP	MPV
45	271	29	0.11	AdaLiftOver	ERY	MPV
46	271	52	0.19	AdaLiftOver	GMP	MPV
47	271	46	0.17	AdaLiftOver	Mega	MPV
48	271	39	0.14	AdaLiftOver	MEP	MPV
49	271	16	0.06	AdaLiftOver	Mono	MPV

Continued on next page

Supplementary Table 14 – continued from previous page

	# mapped	# overlapped	precision	method	cell type	trait
50	271	25	0.09	AdaLiftOver	NK	MPV
51	277	15	0.05	bnMapper	B	MPV
52	277	16	0.06	bnMapper	CD4	MPV
53	277	19	0.07	bnMapper	CD8	MPV
54	277	46	0.17	bnMapper	CMP	MPV
55	277	20	0.07	bnMapper	ERY	MPV
56	277	41	0.15	bnMapper	GMP	MPV
57	277	36	0.13	bnMapper	Mega	MPV
58	277	34	0.12	bnMapper	MEP	MPV
59	277	15	0.05	bnMapper	Mono	MPV
60	277	20	0.07	bnMapper	NK	MPV
61	590	12	0.02	EpiAlignment	B	MPV
62	590	8	0.01	EpiAlignment	CD4	MPV
63	590	9	0.02	EpiAlignment	CD8	MPV
64	590	41	0.07	EpiAlignment	CMP	MPV
65	590	26	0.04	EpiAlignment	ERY	MPV
66	590	47	0.08	EpiAlignment	GMP	MPV
67	590	38	0.06	EpiAlignment	Mega	MPV
68	590	29	0.05	EpiAlignment	MEP	MPV
69	590	20	0.03	EpiAlignment	Mono	MPV
70	590	15	0.03	EpiAlignment	NK	MPV
71	287	16	0.06	UCSC liftOver	B	MPV
72	287	16	0.06	UCSC liftOver	CD4	MPV
73	287	19	0.07	UCSC liftOver	CD8	MPV
74	287	48	0.17	UCSC liftOver	CMP	MPV
75	287	22	0.08	UCSC liftOver	ERY	MPV
76	287	42	0.15	UCSC liftOver	GMP	MPV
77	287	38	0.13	UCSC liftOver	Mega	MPV
78	287	36	0.13	UCSC liftOver	MEP	MPV
79	287	16	0.06	UCSC liftOver	Mono	MPV
80	287	21	0.07	UCSC liftOver	NK	MPV
81	161	14	0.09	AdaLiftOver	B	Mono
82	161	17	0.11	AdaLiftOver	CD4	Mono
83	161	15	0.09	AdaLiftOver	CD8	Mono
84	161	24	0.15	AdaLiftOver	CMP	Mono
85	161	8	0.05	AdaLiftOver	ERY	Mono
86	161	39	0.24	AdaLiftOver	GMP	Mono
87	161	23	0.14	AdaLiftOver	Mega	Mono
88	161	10	0.06	AdaLiftOver	MEP	Mono
89	161	29	0.18	AdaLiftOver	Mono	Mono
90	161	20	0.12	AdaLiftOver	NK	Mono
91	186	14	0.08	bnMapper	B	Mono
92	186	10	0.05	bnMapper	CD4	Mono
93	186	9	0.05	bnMapper	CD8	Mono
94	186	22	0.12	bnMapper	CMP	Mono
95	186	7	0.04	bnMapper	ERY	Mono
96	186	41	0.22	bnMapper	GMP	Mono
97	186	29	0.16	bnMapper	Mega	Mono

Continued on next page

Supplementary Table 14 – continued from previous page

	# mapped	# overlapped	precision	method	cell type	trait
98	186	11	0.06	bnMapper	MEP	Mono
99	186	24	0.13	bnMapper	Mono	Mono
100	186	16	0.09	bnMapper	NK	Mono
101	383	14	0.04	EpiAlignment	B	Mono
102	383	9	0.02	EpiAlignment	CD4	Mono
103	383	15	0.04	EpiAlignment	CD8	Mono
104	383	12	0.03	EpiAlignment	CMP	Mono
105	383	3	0.01	EpiAlignment	ERY	Mono
106	383	26	0.07	EpiAlignment	GMP	Mono
107	383	10	0.03	EpiAlignment	Mega	Mono
108	383	9	0.02	EpiAlignment	MEP	Mono
109	383	20	0.05	EpiAlignment	Mono	Mono
110	383	10	0.03	EpiAlignment	NK	Mono
111	202	16	0.08	UCSC liftOver	B	Mono
112	202	11	0.05	UCSC liftOver	CD4	Mono
113	202	9	0.04	UCSC liftOver	CD8	Mono
114	202	24	0.12	UCSC liftOver	CMP	Mono
115	202	7	0.03	UCSC liftOver	ERY	Mono
116	202	44	0.22	UCSC liftOver	GMP	Mono
117	202	31	0.15	UCSC liftOver	Mega	Mono
118	202	11	0.05	UCSC liftOver	MEP	Mono
119	202	25	0.12	UCSC liftOver	Mono	Mono
120	202	17	0.08	UCSC liftOver	NK	Mono
121	148	31	0.21	AdaLiftOver	B	Lymph
122	148	31	0.21	AdaLiftOver	CD4	Lymph
123	148	33	0.22	AdaLiftOver	CD8	Lymph
124	148	16	0.11	AdaLiftOver	CMP	Lymph
125	148	12	0.08	AdaLiftOver	ERY	Lymph
126	148	16	0.11	AdaLiftOver	GMP	Lymph
127	148	18	0.12	AdaLiftOver	Mega	Lymph
128	148	15	0.10	AdaLiftOver	MEP	Lymph
129	148	13	0.09	AdaLiftOver	Mono	Lymph
130	148	27	0.18	AdaLiftOver	NK	Lymph
131	194	20	0.10	bnMapper	B	Lymph
132	194	23	0.12	bnMapper	CD4	Lymph
133	194	24	0.12	bnMapper	CD8	Lymph
134	194	9	0.05	bnMapper	CMP	Lymph
135	194	7	0.04	bnMapper	ERY	Lymph
136	194	8	0.04	bnMapper	GMP	Lymph
137	194	10	0.05	bnMapper	Mega	Lymph
138	194	11	0.06	bnMapper	MEP	Lymph
139	194	8	0.04	bnMapper	Mono	Lymph
140	194	18	0.09	bnMapper	NK	Lymph
141	412	19	0.05	EpiAlignment	B	Lymph
142	412	20	0.05	EpiAlignment	CD4	Lymph
143	412	19	0.05	EpiAlignment	CD8	Lymph
144	412	13	0.03	EpiAlignment	CMP	Lymph
145	412	5	0.01	EpiAlignment	ERY	Lymph

Continued on next page

Supplementary Table 14 – continued from previous page

	# mapped	# overlapped	precision	method	cell type	trait
146	412	12	0.03	EpiAlignment	GMP	Lymph
147	412	11	0.03	EpiAlignment	Mega	Lymph
148	412	4	0.01	EpiAlignment	MEP	Lymph
149	412	8	0.02	EpiAlignment	Mono	Lymph
150	412	20	0.05	EpiAlignment	NK	Lymph
151	209	22	0.11	UCSC liftOver	B	Lymph
152	209	25	0.12	UCSC liftOver	CD4	Lymph
153	209	26	0.12	UCSC liftOver	CD8	Lymph
154	209	11	0.05	UCSC liftOver	CMP	Lymph
155	209	7	0.03	UCSC liftOver	ERY	Lymph
156	209	9	0.04	UCSC liftOver	GMP	Lymph
157	209	11	0.05	UCSC liftOver	Mega	Lymph
158	209	11	0.05	UCSC liftOver	MEP	Lymph
159	209	10	0.05	UCSC liftOver	Mono	Lymph
160	209	20	0.10	UCSC liftOver	NK	Lymph
161	47	1	0.02	AdaLiftOver	B	Alzheimer
162	47	0	0.00	AdaLiftOver	CD4	Alzheimer
163	47	3	0.06	AdaLiftOver	CD8	Alzheimer
164	47	3	0.06	AdaLiftOver	CMP	Alzheimer
165	47	1	0.02	AdaLiftOver	ERY	Alzheimer
166	47	3	0.06	AdaLiftOver	GMP	Alzheimer
167	47	3	0.06	AdaLiftOver	Mega	Alzheimer
168	47	1	0.02	AdaLiftOver	MEP	Alzheimer
169	47	0	0.00	AdaLiftOver	Mono	Alzheimer
170	47	1	0.02	AdaLiftOver	NK	Alzheimer
171	91	0	0.00	bnMapper	B	Alzheimer
172	91	0	0.00	bnMapper	CD4	Alzheimer
173	91	2	0.02	bnMapper	CD8	Alzheimer
174	91	2	0.02	bnMapper	CMP	Alzheimer
175	91	0	0.00	bnMapper	ERY	Alzheimer
176	91	3	0.03	bnMapper	GMP	Alzheimer
177	91	4	0.04	bnMapper	Mega	Alzheimer
178	91	0	0.00	bnMapper	MEP	Alzheimer
179	91	0	0.00	bnMapper	Mono	Alzheimer
180	91	0	0.00	bnMapper	NK	Alzheimer
181	204	6	0.03	EpiAlignment	B	Alzheimer
182	204	3	0.01	EpiAlignment	CD4	Alzheimer
183	204	0	0.00	EpiAlignment	CD8	Alzheimer
184	204	5	0.02	EpiAlignment	CMP	Alzheimer
185	204	2	0.01	EpiAlignment	ERY	Alzheimer
186	204	6	0.03	EpiAlignment	GMP	Alzheimer
187	204	2	0.01	EpiAlignment	Mega	Alzheimer
188	204	2	0.01	EpiAlignment	MEP	Alzheimer
189	204	1	0.00	EpiAlignment	Mono	Alzheimer
190	204	3	0.01	EpiAlignment	NK	Alzheimer
191	94	0	0.00	UCSC liftOver	B	Alzheimer
192	94	0	0.00	UCSC liftOver	CD4	Alzheimer
193	94	2	0.02	UCSC liftOver	CD8	Alzheimer

Continued on next page

Supplementary Table 14 – continued from previous page

	# mapped	# overlapped	precision	method	cell type	trait
194	94	2	0.02	UCSC liftOver	CMP	Alzheimer
195	94	0	0.00	UCSC liftOver	ERY	Alzheimer
196	94	3	0.03	UCSC liftOver	GMP	Alzheimer
197	94	4	0.04	UCSC liftOver	Mega	Alzheimer
198	94	0	0.00	UCSC liftOver	MEP	Alzheimer
199	94	0	0.00	UCSC liftOver	Mono	Alzheimer
200	94	0	0.00	UCSC liftOver	NK	Alzheimer

Supplementary Table 14: Benchmarking AdaLiftOver on hematopoietic GWAS SNPs for 4 traits: MCV (570), MPV (646), Mono (441), Lymph (466). The Alzheimer (218) GWAS SNPs are treated as negative controls.

	# mapped GWAS	# overlapped GWAS	# genes	precision	method
1	1060	86	30	0.08	AdaLiftOver
2	1502	62	28	0.04	bnMapper
3	1586	65	29	0.04	UCSC liftOver
4	2526	62	23	0.02	EpiAlignment

Supplementary Table 15: Benchmarking AdaLiftOver on BMD GWAS SNPs. Mapping of the 3,125 BMD fine-mapped GWAS SNPs to 52 BMD genes in mouse. Distance = 250 kb.

	# mapped GWAS	# overlapped GWAS	# genes	precision	distance	method
1	29486	14	11	0.00	0	AdaLiftOver
2	38797	5	3	0.00	0	bnMapper
3	41278	6	4	0.00	0	UCSC liftOver
4	29486	1096	71	0.04	100kb	AdaLiftOver
5	38797	791	67	0.02	100kb	bnMapper
6	41278	878	74	0.02	100kb	UCSC liftOver
7	29486	2140	90	0.07	250kb	AdaLiftOver
8	38797	1661	89	0.04	250kb	bnMapper
9	41278	1813	102	0.04	250kb	UCSC liftOver

Supplementary Table 16: Benchmarking AdaLiftOver on BMD GWAS SNPs. Mapping of the 116,402 BMD fine-mapped GWAS SNPs to 200 BMD genes in mouse.

3 Supplementary Figures

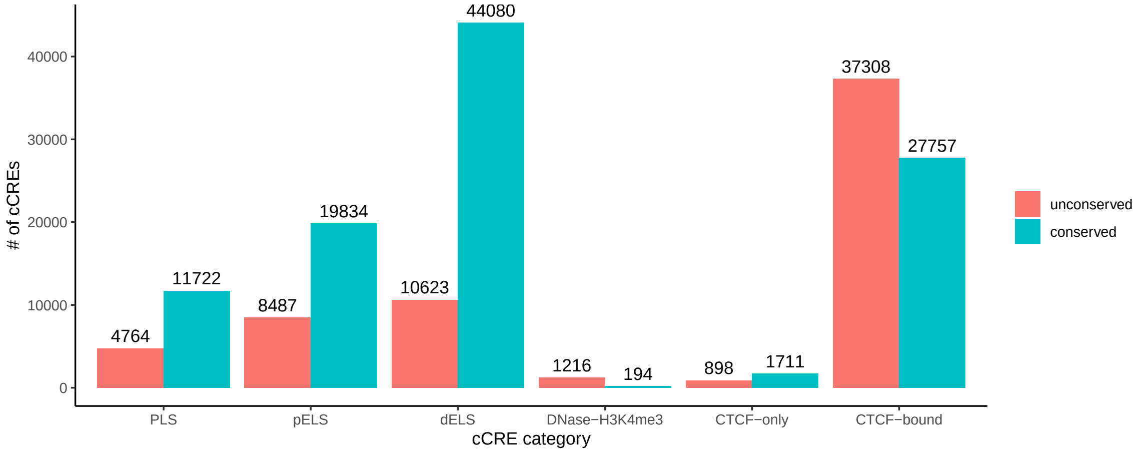

Supplementary Figure 1: The summary of categories between orthologous cCREs. X-axis: the human cCRE category. Y-axis: the number of orthologous mouse cCREs that fall into the same category (conserved) or a different category (unconserved).

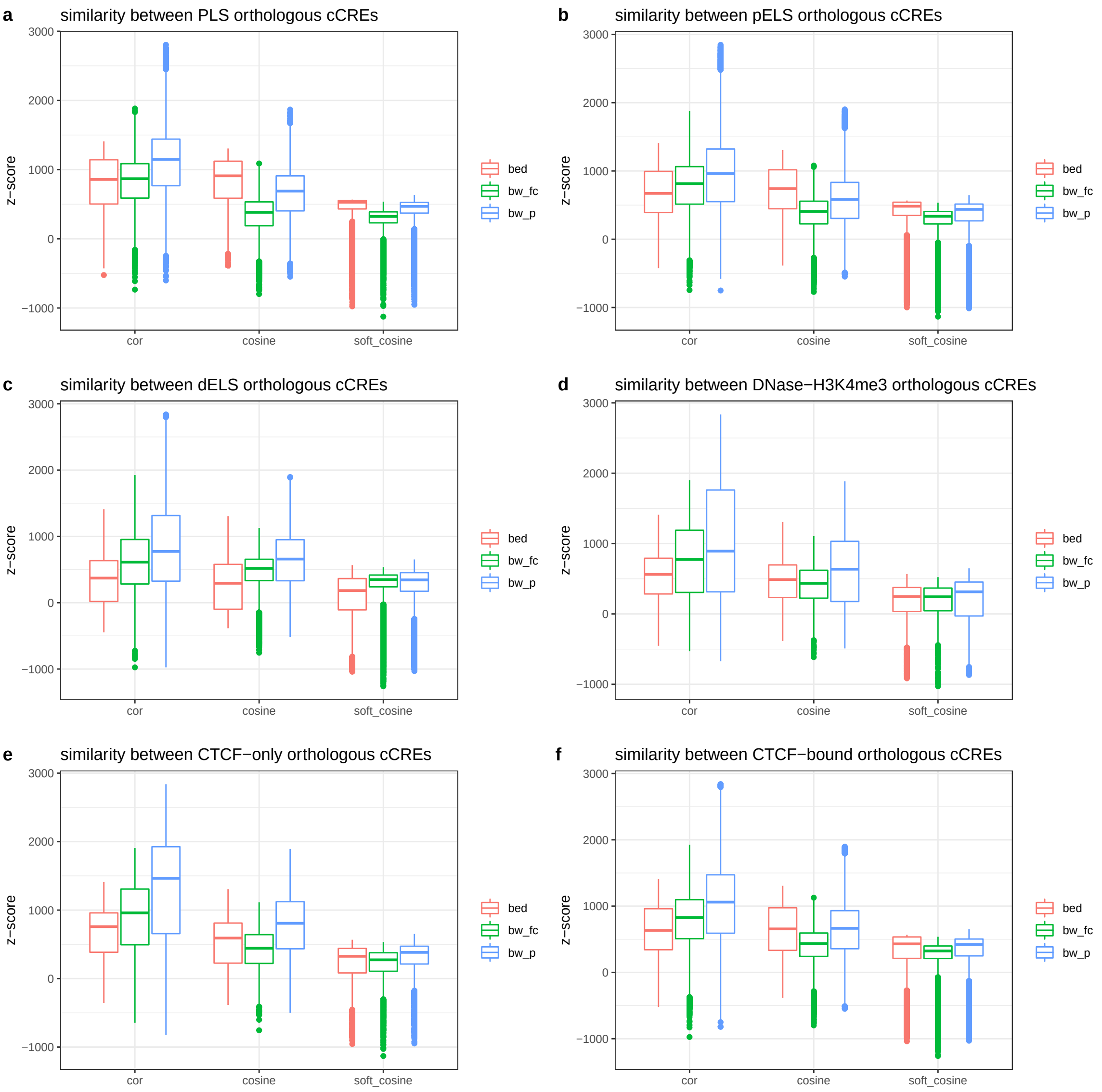

Supplementary Figure 2: Comparison of epigenome similarities for different classes of orthogolous cCREs with three different metrics (cor: Pearson's correlation; cosine: cosine similarity; soft_cosine: soft cosine similarity) and three different data formats (bed: BED format; bw_fc: fold change BigWig format; bw_p: p-value BigWig format) for constructing the epigenomic features. A null distribution for each of the similarity scores is estimated by randomly permuting cCREs 10,000 times. Observed similarity scores were transformed into z-scores using the mean and variance estimates of these null distributions.

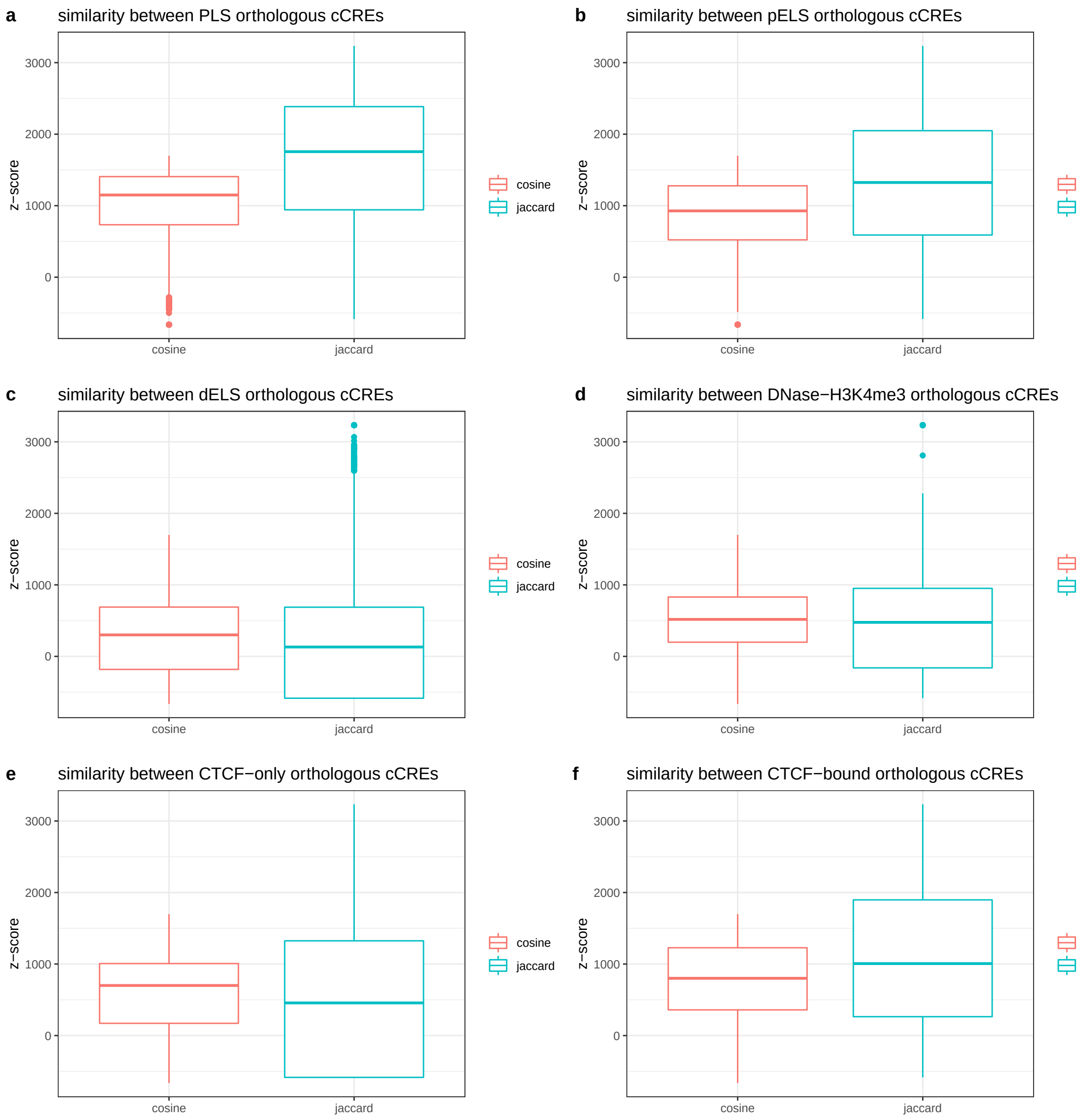

Supplementary Figure 3: Comparison of similarity metrics (cosine: cosine similarity; jaccard: Jaccard similarity) for binary epigenomic features (BED format). A null distribution for each of the similarity scores is estimated by randomly permuting cCREs. Observed similarity scores were transformed into z-scores using the mean and variance estimates of these null distributions.

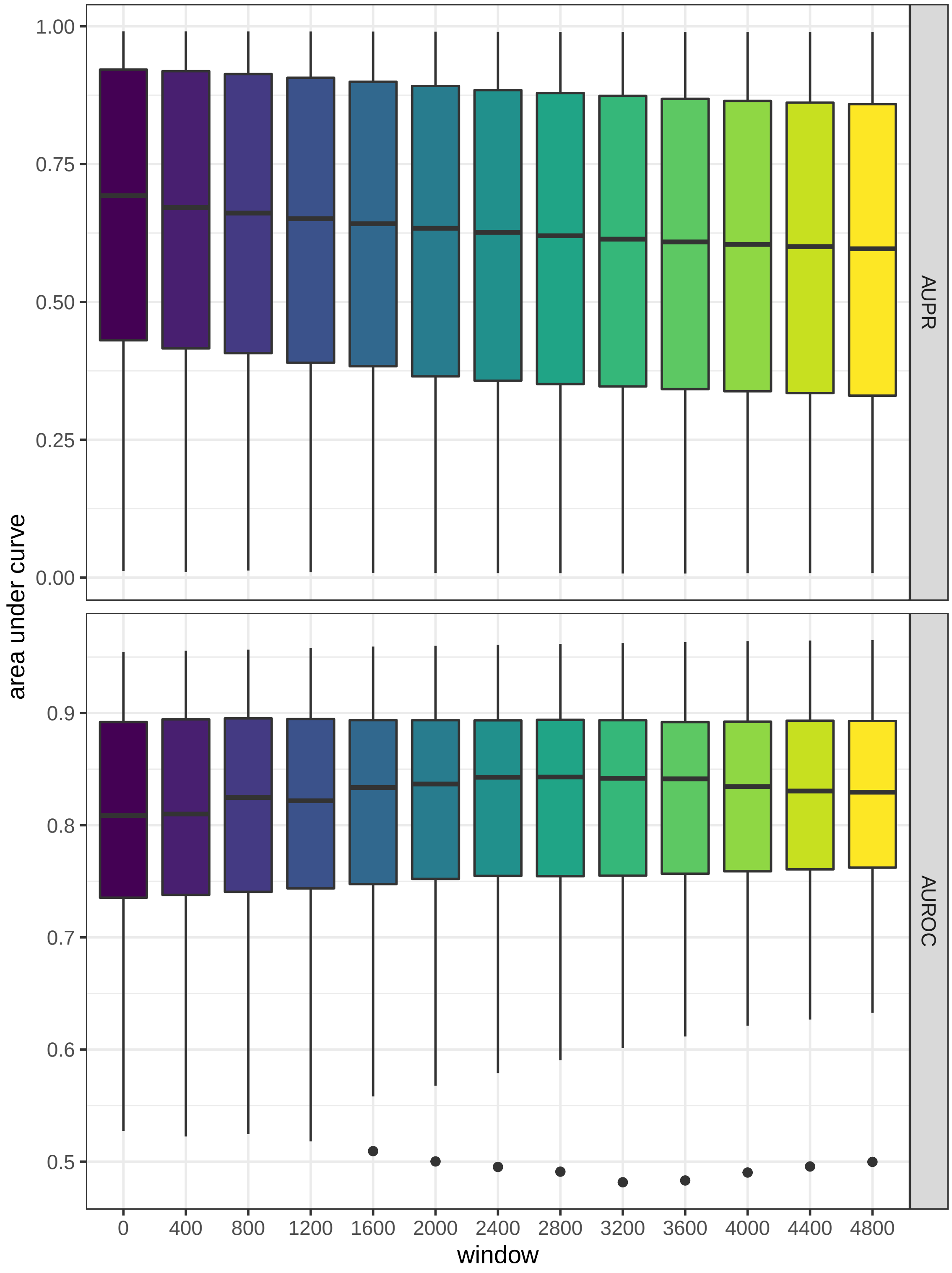

Supplementary Figure 4: Summary of the leave-one-out cross-validation experiments with 67 epigenome datasets over a grid of windows.

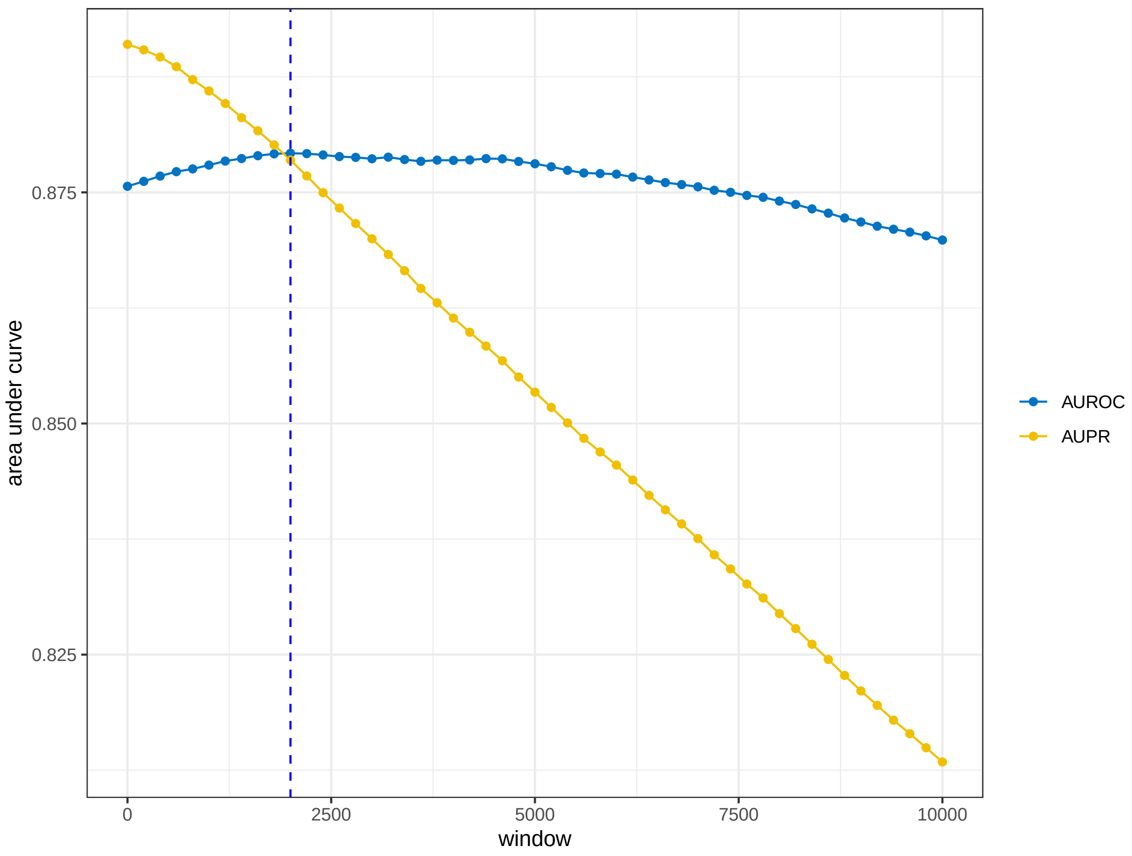

Supplementary Figure 5: The area under ROC/PR curve over a grid of windows from the mouse islet ATAC-seq peak analysis. AUROC and AUPR values are obtained for the set of candidate target regions generated at different local window sizes by fitting a logistic regression on the data labelled using the human islet ATAC-seq peaks as the gold standard. The vertical blue dashed line depicts window size of 2 kb.

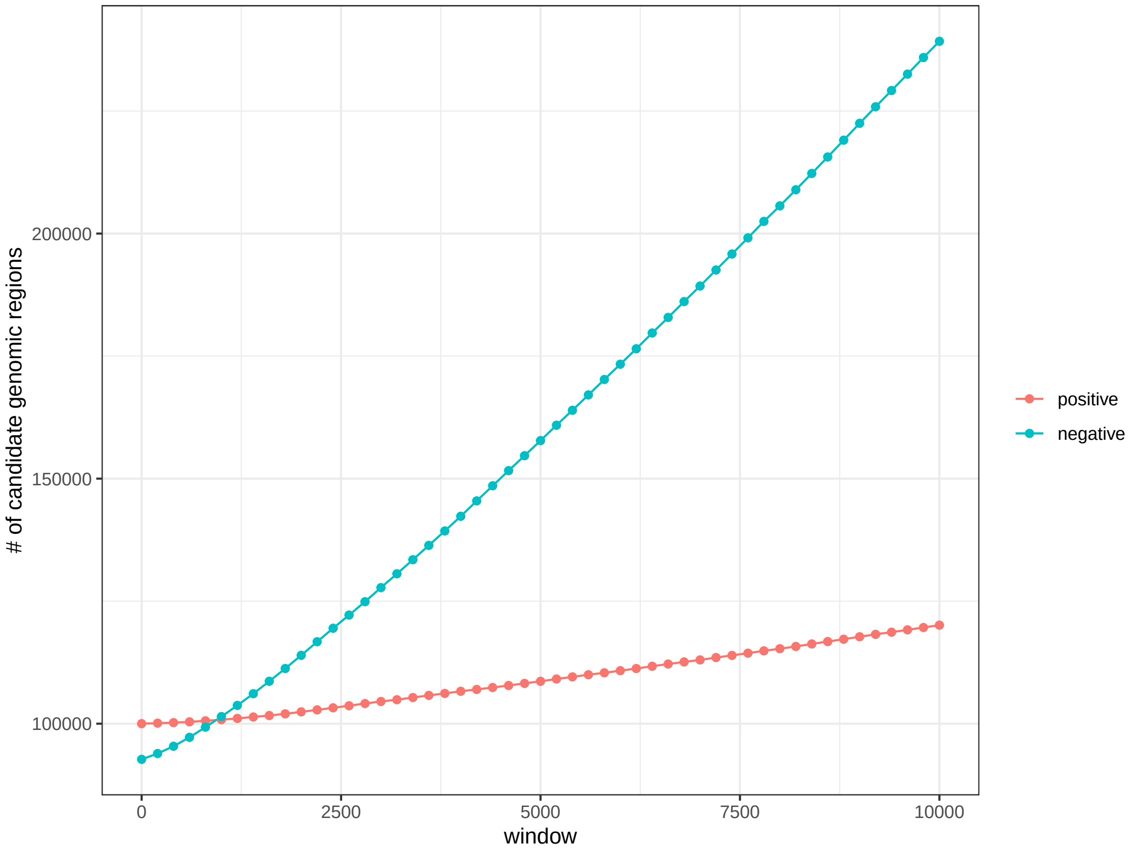

Supplementary Figure 6: As the local window size increases, it generates more candidate target regions, which results in an imbalance between the numbers of positive and negative cases, and hence the decreasing pattern in AUPR (Supplementary Figure 5).

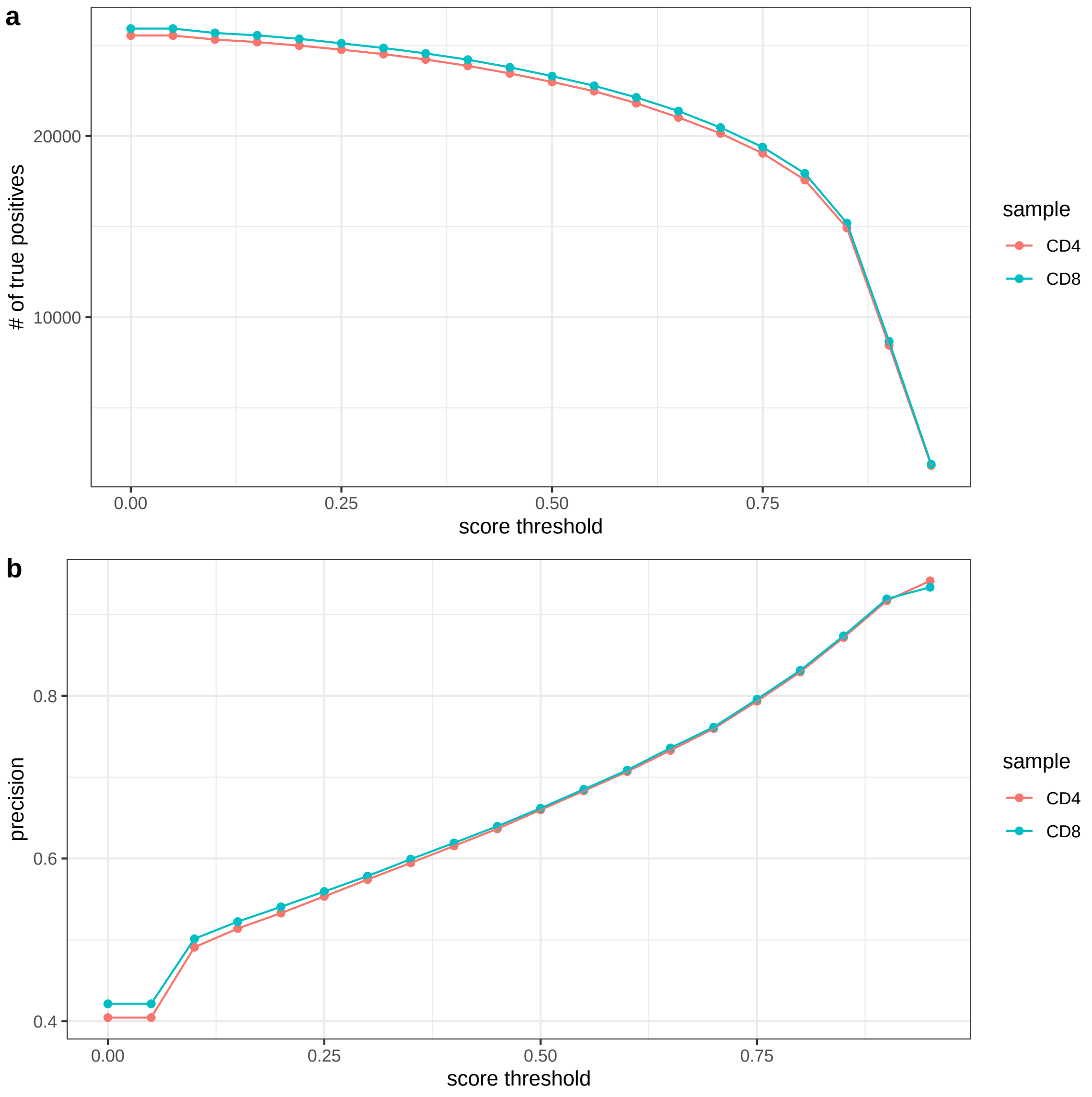

Supplementary Figure 7: The effects of logistic probability score threshold over # of true positives (a) and precision (b) for the orthologous CD4 and CD8 ATAC-seq data. # of true positives: the number of mouse-derived peaks that overlaps human peaks; precision: # of true positives / # of mouse-derived peaks.

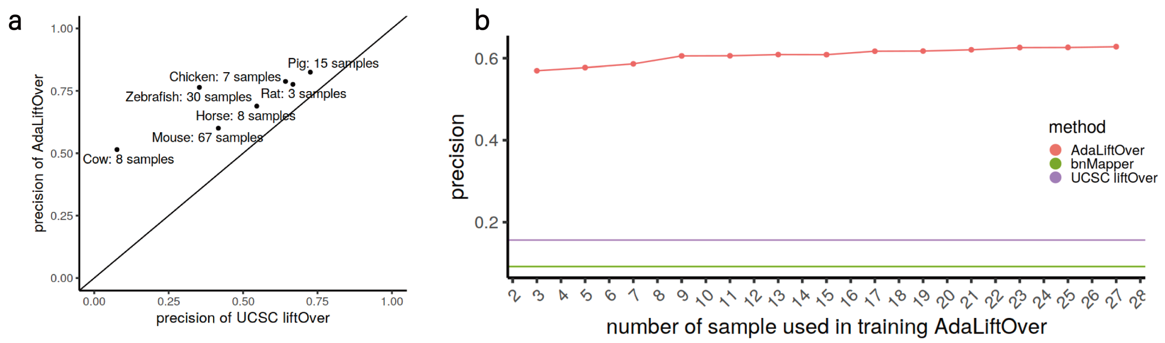

Supplementary Figure 8: **a.** Average precision of AdaLiftOver versus UCSC liftOver across 7 species. **b.** Precision of AdaLiftOver as a function of the number of epigenome datasets used in the training in zebrafish.

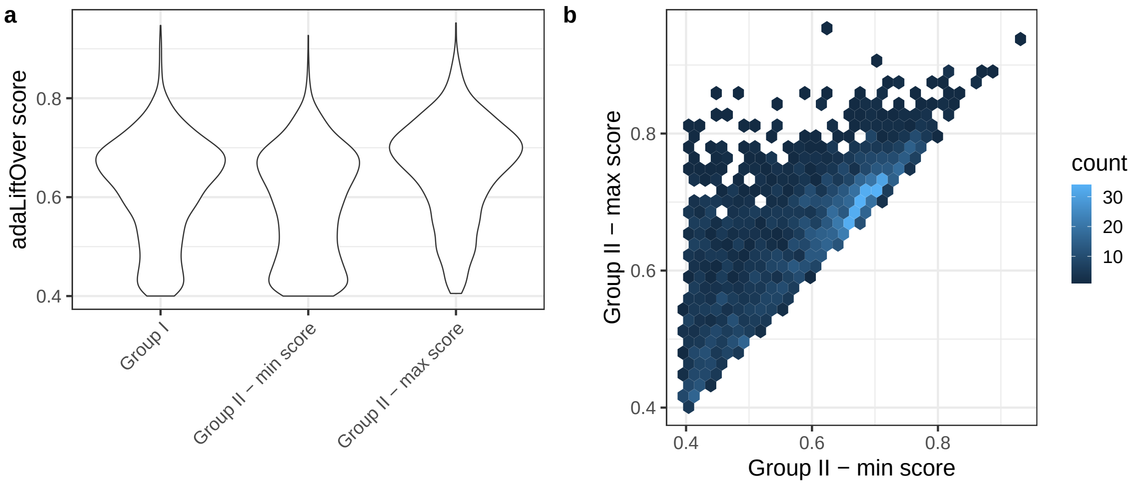

Supplementary Figure 9: **a.** adaLift scores of regions in groups I (unique regions) and II (multi-mapper regions with min and max scores) after filtering regions with scores less than 0.4. **b.** Maximum and minimum adaLiftOver scores for regions in Group II (multi-region mappings) after filtering regions with scores less than 0.4.

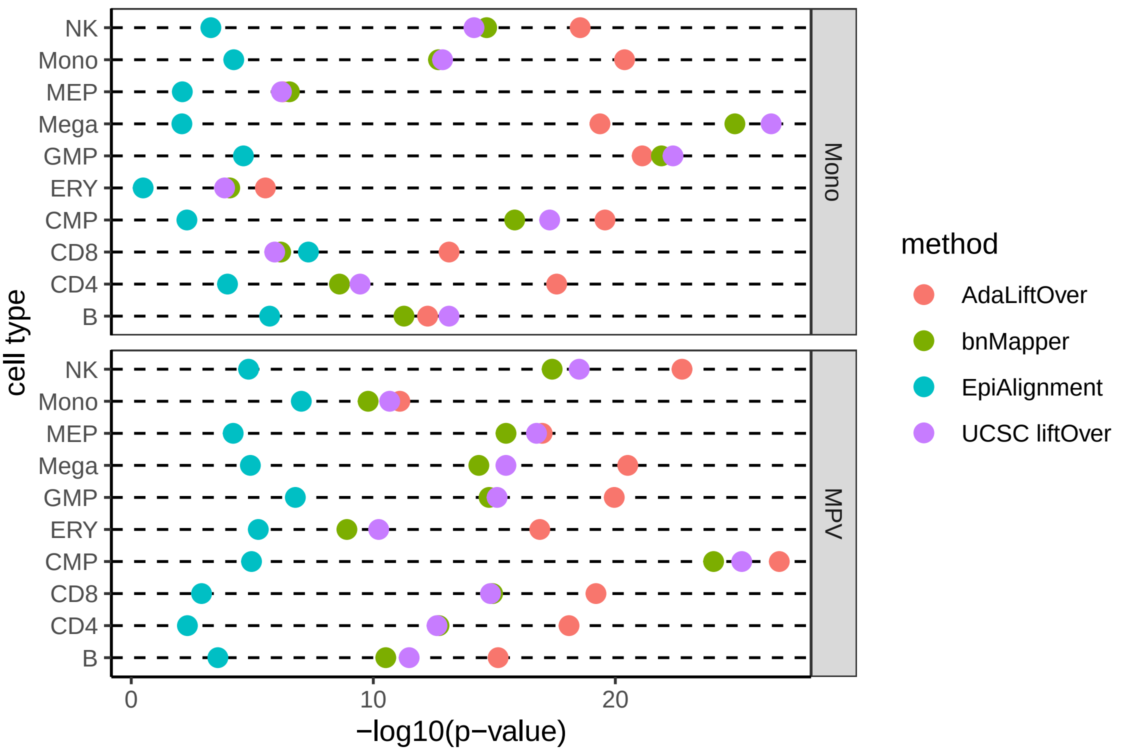

Supplementary Figure 10: Enrichment analysis for fine-mapped GWAS SNPs from two other hematopoietic traits with respect to 10 hematopoietic ATAC-seq peaks. Mono: monocyte count, MPV: mean platelet volume.

References

Cheng, Y., Ma, Z., Kim, B.-H., Wu, W., Cayting, P., Boyle, A. P., Sundaram, V., Xing, X., Dogan, N., Li, J., *et al.* (2014). Principles of regulatory information conservation between mouse and human. *Nature*, **515**(7527), 371–375.

Denas, O., Sandstrom, R., Cheng, Y., Beal, K., Herrero, J., Hardison, R. C., and Taylor, J. (2015). Genome-wide comparative analysis reveals human-mouse regulatory landscape and evolution. *BMC genomics*, **16**(1), 1–9.

Hinrichs, A. S., Karolchik, D., Baertsch, R., Barber, G. P., Bejerano, G., Clawson, H., Diekhans, M., Furey, T. S., Harte, R. A., Hsu, F., *et al.* (2006). The ucsc genome browser database: update 2006. *Nucleic acids research*, **34**(suppl_1), D590–D598.

Hook, P. W. and McCallion, A. S. (2020). Leveraging mouse chromatin data for heritability enrichment informs common disease architecture and reveals cortical layer contributions to schizophrenia. *Genome research*, **30**(4), 528–539.

Kern, C., Wang, Y., Xu, X., Pan, Z., Halstead, M., Chanthavixay, G., Saelao, P., Waters, S., Xiang, R., Chamberlain, A., *et al.* (2021). Functional annotations of three domestic animal genomes provide vital resources for comparative and agricultural research. *Nature communications*, **12**(1), 1821.

Kingsley, N., Kern, C., Creppe, C., Hales, E. N., Zhou, H., Kalbfleisch, T., MacLeod, J. N., Petersen, J. L., Finno, C. J., and Bellone, R. R. (2019). Functionally annotating regulatory elements in the equine genome using histone mark chip-seq. *Genes*, **11**(1), 3.

Lien, Y.-C., Wang, P. Z., Lu, X. M., and Simmons, R. A. (2020). Altered transcription factor binding and gene bivalency in islets of intrauterine growth retarded rats. *Cells*, **9**(6), 1435.

Lu, J., Cao, X., and Zhong, S. (2019). Epialignment: alignment with both dna sequence and epigenomic data. *Nucleic acids research*, **47**(W1), W11–W19.

Morris, J. A., Kemp, J. P., Youlten, S. E., Laurent, L., Logan, J. G., Chai, R. C., Vulpescu, N. A., Forgetta, V., Kleinman, A., Mohanty, S. T., *et al.* (2019). An atlas of genetic influences on osteoporosis in humans and mice. *Nature genetics*, **51**(2), 258–266.

Rintisch, C., Heinig, M., Bauerfeind, A., Schafer, S., Mieth, C., Patone, G., Hummel, O., Chen, W., Cook, S., Cuppen, E., *et al.* (2014). Natural variation of histone modification and its impact on gene expression in the rat genome. *Genome research*, **24**(6), 942–953.

Treviño, L. S., Dong, J., Kaushal, A., Katz, T. A., Jangid, R. K., Robertson, M. J., Grimm, S. L., Ambati, C. S. R., Putluri, V., Cox, A. R., *et al.* (2020). Epigenome environment interactions accelerate epigenomic aging and unlock metabolically restricted epigenetic reprogramming in adulthood. *Nature communications*, **11**(1), 2316.

Ulirsch, J. C., Lareau, C. A., Bao, E. L., Ludwig, L. S., Guo, M. H., Benner, C., Satpathy, A. T., Kartha, V. K., Salem, R. M., Hirschhorn, J. N., *et al.* (2019). Interrogation of human hematopoiesis at single-cell and single-variant resolution. *Nature genetics*, **51**(4), 683–693.

Xiang, G., Keller, C. A., Heuston, E., Giardine, B. M., An, L., Wixom, A. Q., Miller, A., Cockburn, A., Sauria, M. E., Weaver, K., *et al.* (2020). An integrative view of the regulatory and transcriptional landscapes in mouse hematopoiesis. *Genome research*, **30**(3), 472–484.

Yang, H., Luan, Y., Liu, T., Lee, H. J., Fang, L., Wang, Y., Wang, X., Zhang, B., Jin, Q., Ang, K. C., *et al.* (2020). A map of cis-regulatory elements and 3d genome structures in zebrafish. *Nature*, **588**(7837), 337–343.

Zhao, Y., Hou, Y., Xu, Y., Luan, Y., Zhou, H., Qi, X., Hu, M., Wang, D., Wang, Z., Fu, Y., *et al.* (2021). A compendium and comparative epigenomics analysis of cis-regulatory elements in the pig genome. *Nature communications*, **12**(1), 2217.
